# mTOR promotes the formation and growth of tertiary lymphoid tissues in the kidney

**DOI:** 10.3389/fimmu.2025.1527817

**Published:** 2025-05-27

**Authors:** Daniel J. Atwood, Zhibin He, Makoto Miyazaki, Katharina Hopp, Alkesh Jani, Seth B. Furgeson, Sarah Faubel, Charles L. Edelstein

**Affiliations:** ^1^ Renal Division, Rocky Mountain Regional Veteran’s Affairs (VA) Medical Center, Aurora, CO, United States; ^2^ Division of Renal Diseases and Hypertension, University of Colorado Anschutz Medical Campus, Aurora, CO, United States

**Keywords:** autophagy, mTOR, polycystic kidney, p62, tertiary lymphoid tissue

## Abstract

Tertiary lymphoid tissues (TLTs) are ectopic lymphoid tissues that form *de novo* in nonlymphoid organs. In this study, we demonstrate that the kidneys of aged mice with a renal tubule-specific knockout of autophagy-related 7 (Atg7) contain numerous and large TLTs. p-S6 protein, a marker of mTORC1, was elevated in the tubules adjacent to the TLTs as well as within the TLTs themselves. In Atg7^−/−^ kidneys, tubular injury and increased proinflammatory cytokines were observed, both of which are known to promote TLT formation and growth. In mice with either polycystic kidney disease (Pkd1^RC/RC^) or kidney ischemia, increased p-S6 was observed in tubules near TLTs and within the TLTs. Treatment with Torin2, an mTOR inhibitor, led to the virtual disappearance of TLTs in Pkd1^RC/RC^ kidneys and a significant reduction in TLTs in ischemic kidneys. To assess whether p-S6 in the tubules was driving TLT formation, ischemia was induced in tubule-specific Atg7^−/−^ Raptor (mTORC1)^−/−^ mice. The tubule-specific Raptor knockout had little effect on the TLTs. In summary, Torin2, which inhibited p-S6 in both tubules and TLTs, resulted in a large decrease in TLTs in ischemic and Pkd1^RC/RC^ kidneys. Tubule-specific knockout of mTORC1 (Raptor) had no effect on TLTs. In conclusion, p-S6 activity within the TLTs, rather than in the tubules, drives the proliferation of immune cells and the formation and growth of TLTs. These findings provide new insights into the role of mTOR in TLT development. The study has important therapeutic implications, as TLTs are involved in numerous disease processes and mTOR inhibitors are widely used in clinical practice.

## Introduction

The primary lymphoid organs are the bone marrow, where blood and immune cells are produced, and the thymus, where T-lymphocytes mature. The main secondary lymphoid organs are lymph nodes and the spleen. Tertiary lymphoid tissues (TLTs), also known as tertiary lymphoid organs (TLOs) or TLSs, are ectopic lymphoid tissues composed of lymphocyte aggregates that develop *de novo* in nonlymphoid organs. TLTs have been understudied due to the absence of a clear definition, lack of standardized detection methods, and the absence of *in vivo* mouse models or *in vitro* systems that recapitulate TLT formation (1). However, recent studies have clarified the developmental stages of TLT formation in mouse and human kidneys and explored the structural and functional characteristics that define TLTs (2).

Important evidence is emerging that TLTs play a role in the severity, response to therapy, and prognosis of cancer, infection, autoimmune diseases, transplant rejection, and chronic inflammatory diseases (1). TLTs have been found in the kidneys of patients with glomerulonephritis (3), lupus nephritis (4), vasculitis (5), pyelonephritis (2), and in transplanted kidneys (6). In transplanted kidneys, the presence of TLTs is associated with chronic allograft rejection (6). TLTs have been detected in the brain in patients with SLE and in synovium, lung, and bone marrow in rheumatoid arthritis (7). Mature tertiary lymphoid structures predict immune checkpoint inhibitor efficacy in solid tumors (8). TLTs form in the lung in viral and bacterial infections, asthma, and chronic obstructive pulmonary disease (COPD) (9). Spontaneous age-dependent TLT formation has been observed in the liver and bladder (10). A better understanding of the mechanism of formation and growth of TLTs will have important implications in understanding the role of TLTs in chronic disease and in developing future therapies. Therapeutic interventions that target the formation and growth of TLTs may potentially be beneficial in chronic kidney disease, autoimmune diseases, and other chronic inflammatory diseases.

Polycystic kidney disease is the most common hereditary kidney disease in which cysts form in the kidney, ultimately leading to kidney failure (11). As suppressed autophagy is thought to play a mechanistic role in cyst growth in polycystic kidney disease (12), the original hypothesis of the present study was that aged autophagy-related 7 knockout (Atg7)^−/−^ kidneys would become cystic. To our surprise, instead of cysts, there were impressive TLTs in aged Atg7^−/−^ kidneys.

As mTORC1 is known to be both upstream and downstream of autophagy (13), we stained the Atg7^−/−^ kidneys for p-S6 to determine the effect of autophagy knockout in the tubules on mTORC1. While there was increased p-S6 staining in the tubules, we were very impressed by the intense p-S6 staining in the TLTs. The finding of intense p-S6 staining in the TLTs in Atg7^−/−^ kidneys led us to further investigate the mechanistic role of mTORC1 in TLT formation and growth. An unresolved question is what molecular pathways drive the proliferation of cells within TLTs, resulting in the initiation, maturation, and maintenance of TLTs.

Most studies have focused on what drives the proliferation of individual immune cells within the TLTS. For example, the B-cell-activating factor (BAFF) was found to drive the proliferation of B cells in TLTs (14). TNF-superfamily signaling drives the proliferation of unique lymphocyte populations in TLTs (15). TLT-associated fibroblasts exhibit signal transducer and activator of transcription-1 (STAT-1) activation and produce chemokines like CXC motif ligands 9 and 10 (CXCL9/10) (16). Intense proliferation of cells within the TLTs is a defining characteristic of TLTs (1, 18). The present study focused on the mTORC1 signaling pathway, which is one of the most important and potent drivers of cellular proliferation of multiple cell types that are known to make up the TLTs (17, 19–21). Thus, the hypothesis was developed that mTORC1 in TLTs plays a role in TLT formation and growth. The aim of the study was to determine the mechanistic role of mTORC1 in TLT formation and growth, using pharmacological and genetic techniques, in Atg7 knockout kidneys and in two common models of kidney disease, polycystic kidney disease (PKD) and kidney ischemia.

## Methods

### Study approval

All experiments were performed following the guidelines in the National Institutes of Health Guidelines for the Care and Use of Laboratory Animals. The animal protocol was approved by the Institutional Animal Care and Use Committee of the University of Colorado Anschutz Medical Campus (Protocol No. 00063). Mice were maintained on a standard diet under pathogen-free housing conditions, with food and water available *ad libitum*.

### Atg7^−/−^ mice

Renal tubule-specific cadherin Cre Atg7^−/−^ mice (KSP1.3 Cad Cre Atg7^−/−^ mice) were developed by Cre recombinase technology by breeding KSP 1.3 Cad Cre mice with Atg7 floxed mice. KSP1.3 Cad Cre Atg7^−/−^ mice have expression of Cre recombinase in adult mice restricted to renal tubular epithelial cells with the highest expression in collecting ducts and loops of Henle and low expression in proximal tubules (22, 23). The mean age of the wild-type and Atg7^−/−^ mice used in the current study was 400 days old.

### Regulatory-associated protein of mTOR (Raptor), Atg7 double knockout mice

Renal tubule-specific cadherin Cre Atg7^−/−^, Raptor^−/−^ mice (KSP1.3 Cad Cre Atg7^−/−^, Raptor^−/−^ mice) were developed by Cre recombinase recombination by breeding KSP 1.3 Cad Cre mice with Atg7 floxed mice and Raptor floxed mice.

### Pkd1^RC/RC^ mice

Pkd1^RC/RC^ mice have a hypomorphic *Pkd1* gene mutation orthologous to the PKD patient disease variant, *PKD1* p. R3277C (24). Pkd1^RC/RC^ mice in the C57BL/6 background have cysts at 3 months of age (25). Cyst expansion and size correlate with increased tubular cell proliferation (24). Wild-type C57BL/6J mice (No. 000664) were purchased from Jackson Laboratories (Bar Harbor, ME, USA). Kidneys from our previously published Torin2-treatment study (26) were examined for TLTs.

### *In vivo* studies in Pkd1^RC/RC^ mice

Torin2 is a potent mTOR inhibitor (27). Male and female C57BL/6 Pkd1^RC/RC^ mice were treated with the mTOR inhibitor Torin2 (10 mg/kg IP, daily on weekdays) or vehicle (28% DMSO in PEG300) from days 50 to 120 of age (26). There were equal numbers of males and females per group. Kidneys from our previously published study were used for the present study (26). Torin2 was purchased from ChemieTek, Indianapolis, IN, USA.

Animals received fast imaging with steady-state precession (FISP) abdominal MRI measurements by UC Denver small animal imaging core 1 month before killing, as we have described (28).

### *In vivo* studies: renal ischemia

Six-month-old male wild-type C57BI/6J mice obtained from Jackson Laboratories were used. The ischemic injury was induced by clamping of the left renal pedicle for 45 min. Due to the prolonged time of the clamp, kidneys that did not reperfuse well (returned to pink color) were removed from the studies. The contralateral nonclamped right kidney was used as the control. Mice were killed 45 days after the renal pedicle clamp. The protocol for *in vivo* ischemic injury has previously been detailed by us (29), and the kidney phenotype of 45 min of unilateral renal ischemia in 6-month-old mice has been detailed (30). Mice were treated with the mTOR inhibitor Torin2 (10 mg/kg IP, daily on weekdays) or vehicle (28% DMSO in PEG300) for 35 days, starting from day 10 after the renal pedicle clamp until they were killed. Torin 2 was administered on day 10 after the ischemic insult because it caused mortality if administered in the first week after the ischemic insult when the mice were still recovering from the surgery. Torin2 treatment was started as early as possible after ischemia, as we wanted to determine whether mTOR inhibition could inhibit both the formation and growth of TLTs.

### Dosage of Torin2

In a previous *in vivo* study, we demonstrated that Torin2 at a dose of 10 mg/kg/day IP significantly decreases p-S6 in the kidney, slows cyst growth, and improves kidney function in the Pkd1^RC/RC^ mouse model of ADPKD (26). Thus, the Torin2 dose of 10 mg/kg/day IP was chosen for the current study.

### Measurement of kidney function

Blood urea nitrogen (BUN) was measured with a BioAssay Systems (Hayward, CA, USA) Urea Assay Kit according to the manufacturer’s instructions (DIUR-100). Serum creatinine was measured with high-performance liquid chromatography (HPLC) tandem mass spectrometry. [^2^H3] and creatinine were detected in multiple reaction-monitoring modes, examining transitions of *m*/*z* from 114 to 44.2 and from 117 to 47.2, respectively (31).

### Transcutaneous glomerular filtration rate

Transcutaneous glomerular filtration rate (GFR) (tGFR) was measured and performed as we have previously reported (32). The NIC-Kidney device (MediBeacon Inc, Amtsgericht, Germany) was utilized for tGFR measurements per the manufacturer’s instructions.

### Acute tubular necrosis score

Histological changes due to acute tubular necrosis (ATN) score were evaluated in the outer stripe of the outer medulla on hematoxylin–eosin stained tissue and were quantified by counting the percent of tubules that displayed cell necrosis, loss of brush border, and cast formation as follows: 0 = none, 1 ≤ 10%, 2 = 10%–25%, 3 = 26%–45%, 4 = 46%–75%, and 5 ≥ 75%. At least 10 fields (× 200) were reviewed for each slide.

### Immunoblot analysis

Protein was isolated from tissues using radioimmunoprecipitation assay (RIPA), cOmplete protease, and phoSTOP phosphatase inhibitor cocktails (Sigma, St Louis, MO). Homogenates were centrifuged, and the supernatant was taken for protein quantification by BioRad (Hercules, CA, USA) DC Protein Assay as described by the manufacturer. Samples were mixed with Laemmli sample buffer and boiled for 5 min. Samples were run on 12% fresh polyacrylamide gels. Proteins were then transferred to 0.45 µm PVDF membranes, blocked with 2% evaporated milk, and probed with antibodies listed in **Supplementary Table S1**. The specificity of each of the antibodies used has been validated by the vendor (Cell Signaling Technology, Danvers, MA, USA) and has been cited in our and others’ previous publications (33–39). Blots were developed by chemiluminescence and analyzed for densitometry using ImageJ.

### Routine histology

Tissues were fixed overnight in 10% formalin at 4°C. They were transferred to fresh 70% ethanol and left overnight at 4°C; this process was repeated twice more. Next, the tissues were processed and embedded in paraffin wax using Leica systems. Tissues were sectioned at 4 µm and baked at 60°C for 2 h. Kidneys were stained with hematoxylin–eosin and proliferating cell nuclear antigen (PCNA). TLT number, area, and index (% of the kidney) were quantified in PCNA-stained kidney sections using an NIS Element macro, as previously published (25), in tissue sections visualized by the Aperio ImageScope (Leica Biosystems, St louis, MO).

### Immunohistochemistry protocol

Tissue sections were deparaffinized and rehydrated, then antigen unmasking was performed in sodium citrate buffer (pH 6.0) for 25 min at 100°C. After cooling sections to room temperature in citrate buffer, endogenous peroxidase activity was blocked by immersing the sections in 3% hydrogen peroxide for 10 min, followed by a 5-min rinse in deionized water. Blocking was performed using Vectastain^®^ Elite^®^ ABC Kit blocking serum for 30 min at room temperature. Primary antibodies were diluted in tris-buffered saline with Tween20 (TBST) as indicated in **Supplementary Table S1** and incubated overnight at 4°C in a dark humidified chamber. Immunoreactions were detected using the Vectastain^®^ standard protocol with 3,3′-diaminobenzidine tetrahydrochloride hydrate (DAB) counterstained with hematoxylin. Slides were subsequently dehydrated and mounted. DAB-positive staining was analyzed using Aperio ImageScope-provided macros. Multiplexed staining for cluster of differentiation 45 (CD45), CD4, CD8, SH2 domain-containing leukocyte protein of 76 kDa (SLP76), forkhead box protein P3 (FOXP3), and programmed death ligand 1 (PDL1) was performed by the Human Immune Monitoring Shared Resource Core at the University of Colorado Anschutz using the Vectra Polaris Imaging System (Akoya Biosciences, Malborough, MA) according to manufacturer’s instructions.

### Quantitation of IHC staining

Quantitation of staining was performed using the Aperio ImageScope, Leica Biosystems, by an observer blinded to the treatment modality. The TLT number was counted from the whole kidney stained with PCNA. The TLT index was calculated by dividing the total area of TLTs by the total area of the kidney. For quantitation of TLT staining of proteins, other than PCNA, the percentage of area of the TLT was calculated using the provided Aperio algorithms. As PCNA is a nuclear stain, the percentage of positive cells in the TLT was calculated using the provided Aperio algorithms. For tubular staining, the percentage of positive cells was calculated by randomly selecting 10–15 areas, each 9,000 µm^2^, and measuring positive staining intensity using the provided Aperio algorithms.

### Second-harmonic generation and two-photon excitation fluorescence microscopy

Kidney tissues were fixed in 10% phosphate-buffered formalin (Fisher Scientific, Waltham, MA, USA) and embedded in paraffin. Sections (5 μm thick) were scanned in 15 random regions of interest. Images were acquired at × 20 magnification using a laser-scanning confocal microscope (Zeiss 780, Carl Zeiss, Jena, Germany) equipped with a titanium-sapphire laser (Chameleon Ultra II, Coherent, Santa Clara, CA, USA). The average laser power of 7% at 800 nm (tuned for a second-harmonic generation [SHG]) with 140-fs pulse duration and 80-MHz repetition rate was used. After passage through the microscope optics, the pulse duration was ~ 300 fs. SHG signal was detected on a nondescanned detector following transmission through a filter cube containing a narrow-band 390- to 410-nm emission filter (product no. hq400/20m-2p, Chroma Technology, Bellows Falls, VT, USA). Collagen was quantified using ImageJ (National Institutes of Health). The green (autofluorescence) and red (fibrillar collagens) channels were separated and a threshold was set for the collagen. The percent area was quantified using the threshold value.

### Statistical analysis

Student’s *t*-test was used for comparisons between two independent groups. Multiple group comparisons were performed using analysis of variance (ANOVA) with posttest according to Newman–Keuls. Student’s *t*-test was used for comparisons between two independent groups. A *p*-value of < 0.05 was considered statistically significant. Values are expressed as means ± SEM.

## Results

### TLTs form in the kidney in aged Atg7^−/−^ mice

**Figure 1:**
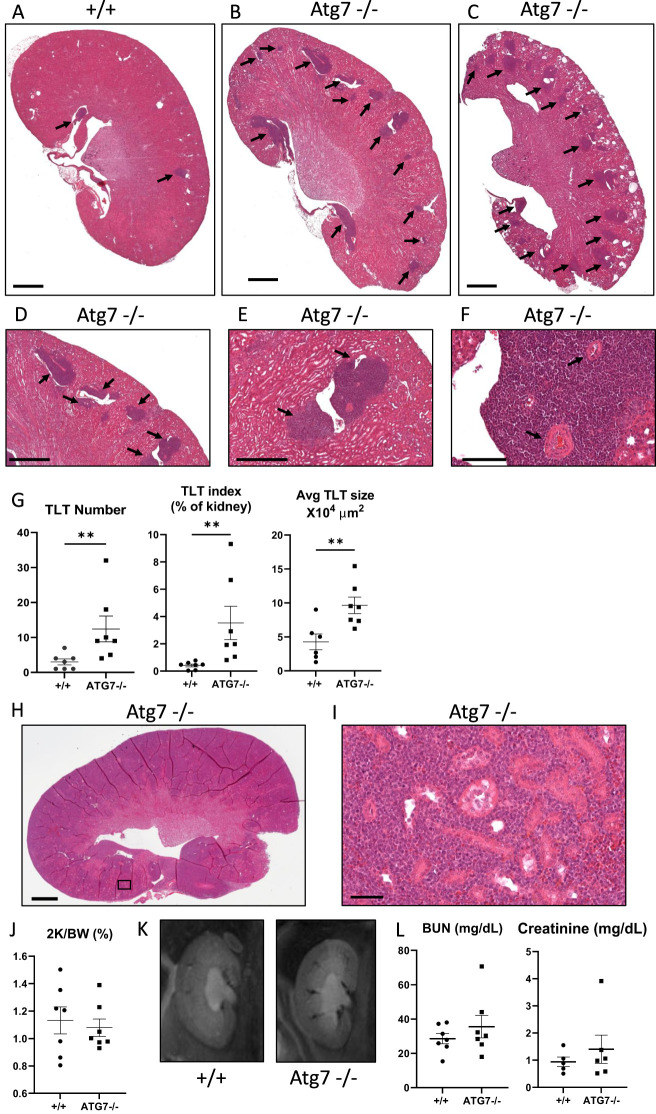
TLTs form in the kidney of aging Atg7^−/−^ mice. Low-power magnification hematoxylin–eosin staining of **(A)** wild-type control kidneys and **(B)** Atg7^−/−^ kidneys. One out of seven Atg7^−/−^ kidneys and no wild-type kidneys were cystic **(C)**. TLTs are indicated by arrows. Higher-power magnifications of hematoxylin–eosin staining of a TLT in the kidney **(D–F)**. Cells within the TLT resembled immune cells **(F)**. TLTs were often located next to blood vessels or completely engulfed blood vessels (arrows) **(F)**. Quantitation of the number of TLTs, TLT index (% of the kidney), average TLT size, and total TLT area was performed on midline longitudinal kidney sections **(G)**. One Atg7^−/−^ kidney was almost virtually replaced by lymphoid tissue (blue staining) **(H)**. Higher-power staining of the kidney in **(H)** shows lymphocytes densely packed between the tubules of the Atg7^−/−^ kidney **(I)**. There was no difference in kidney weight **(J)**, MRI appearance **(K)**, or kidney function **(L)** between Atg7^−/−^ and wild-type control (+/+) kidneys. Student’s *t*-test was used for comparisons between two independent groups. A *p*-value of < 0.05 was considered statistically significant. Values are expressed as mean ± SEM. *^p^* < 0.05; ^**^ *p* < 0.01. Scale bar = 1,000 µm **(A–C, H)**. Scale bar = 100 µm **(D–F)**. Scale bar = 50 µm **(I)**.

The striking feature of 400-day-old renal tubule-specific cadherin Cre Atg7^−/−^ mice (KSP1.3 Cad Cre Atg7^−/−^ mice), a conditional Atg7 knockout mouse model, was the presence of large TLTs. On low-power magnification of hematoxylin–eosin-stained kidneys, the number and size of TLTs in kidneys were increased in Atg7^−/−^ kidneys vs. wild-type control kidneys (**Figures 1A, B**). As suppressed autophagy and increased mTOR play a role in kidney cyst growth in polycystic kidney disease (12, 36, 40, 41), our original hypothesis was that Atg7^−/−^ kidneys would be cystic. Only one out of seven Atg7^−/−^ kidneys (**Figure 1C**) and no wild-type kidneys were cystic. On higher power magnification of hematoxylin-eosin staining of a TLT in the kidney, the majority of cells resembled immune cells (**Figures 1D, E**). TLTs were mostly situated next to blood vessels and often engulfed blood vessels (**Figure 1F**). The number, percent of kidney area (TLT index), and average TLT size were increased in Atg7^−/−^ vs. wild-type kidneys (**Figure 1G**). In one Atg7^−/−^ kidney, the TLTs occupied 9% of the area of the kidney (**Figure 1G**). One Atg7^−/−^ kidney was virtually completely replaced by lymphoid tissue (**Figures 1H, I**). Due to the extreme nature of this kidney, it was not included in the analysis in **Figure 1G**.

There was no difference in the kidney weight (**Figure 1J**), the appearance of the kidney on fast imaging with steady-state free precession (FISP)-MRI scan (**Figure 1K**), or kidney function (**Figure 1L**) in aged Atg7^−/−^ vs. wild-type control (+/+) kidneys.

In aged wild-type control (+/+) kidneys there was no difference in the mean TLT number ± SEM (2.0 ± 1 vs. 3.0 ± 1.4) or mean TLT index ± SEM (0.3 ± 0.1 vs. 0.46 ± 0.2) in males versus females. In Atg7^−/−^ kidneys, 6/7 mice were female, so a comparison between males and females was not performed.

### Suppressed autophagy and large increase in p62 in the kidney in Atg7^−/−^ mice

Tubule-specific Atg7^−/−^ mice were expected to have suppressed autophagy in tubular epithelium since Atg7 is an essential enzyme in the process of autophagy. The next aim of the study was to confirm that Atg7 was knocked out in the tubules in the aged renal tubule-specific cadherin Cre Atg7^−/−^ mice (KSP1.3 Cad Cre Atg7^−/−^ mice), a conditional knockout mouse model. During autophagy, the phospholipid phosphatidylethanolamine (PE) (42) anchors LC3-I (microtubule-associated protein 1A/1B-light chain 3) to the emerging phagophore membrane where it is lipidated to form LC3-II. LC3 lipidation is a multistep process, driven by the E1‐like enzymatic activity of homodimeric Atg7. Sequestosome 1/SQSTM1 (p62) is an adapter protein and a cargo receptor for autophagy (43, 44). p62 interacts with phagosomes by binding to LC3 through the LC3-interacting (LIR) domain delivering ubiquitinated cargoes to the autophagosome (45). When autophagosome/lysosome fusion is impaired, then p62 accumulates proportionally to the impairment (43). Thus, in Atg7^−/−^ kidneys, it was expected that there would be a block in the conversion of LC3-I to LC3-II and a build-up of LC3-I and p62 that is bound to LC3-I. On immunoblot analysis, there was a decrease in Atg7, an increase in LC3-I, and a massive increase in p62 in Atg7^−/−^ kidneys (**Supplementary Figures S1A, B**) but not in heart or liver (**Supplementary Figure S1C**), confirming the kidney-specific knockout of autophagy. As the KSP1.3 Cad Cre Atg7^−/−^ mice have an expression of Cre recombinase that is mainly present in distal tubules (medulla) with less expression in proximal tubules (cortex) (22) (23), a complete loss of Atg7 was not expected in immunoblot analysis of whole kidney extracts.

To localize p62 in the Atg7^−/−^ kidneys, immunohistochemistry (IHC) was performed. p62 expression was expected to correlate with the location of Cre recombinase expression in Atg7^−/−^ mice that are highest in collecting ducts and loops of Henle (that are known to be situated mostly in the medulla) and lowest in proximal tubules (known to be situated in the cortex). In +/+ control mice, staining for p62 was not present in the cortex or the medulla (**Supplementary Figure S1D**). As expected, in Atg7^−/−^ mice, staining for p62 was maximal in the medulla of the kidney with less staining in the cortex (**Supplementary Figure S1E**). In Atg7^−/−^ mice, staining for p62 was not seen in the TLTs, but was seen in tubules surrounding TLTs (**Supplementary Figure S1F**).

### TLTs stain positive for TLT markers, immune cells, and p-S6

**Figure 2:**
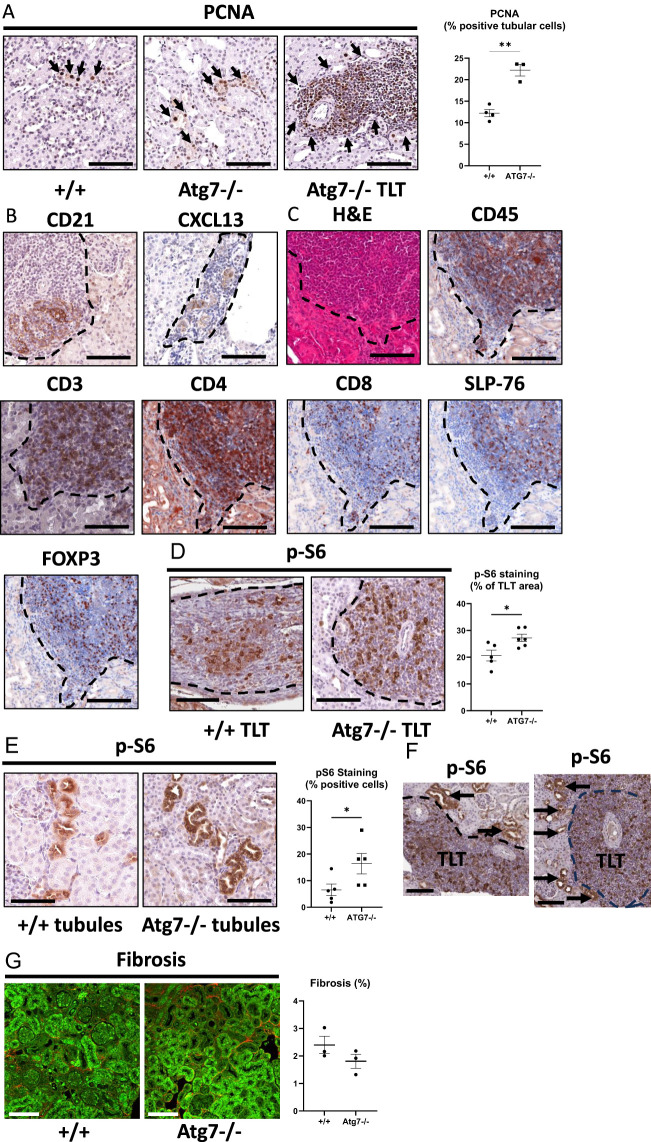
TLTs stain positive for TLT markers PCNA, CD21 CXCL13, and T-cell markers. Minimal fibrosis was observed in the interstitium surrounding TLTs in Atg7^−/−^ kidneys. A representative image and quantitation of PCNA (a proliferation marker) staining in tubules and TLTs, showing PCNA (proliferation) (brown) **(A)**. Representative images of staining for CD21 (follicular dendritic cells), and CXCL13 (chemokine produced by fibroblasts) in TLTs **(B)**. Representative images of IHC staining of TLT for hematoxylin–eosin, CD45 (marker of all hematopoietic cells except for mature erythrocytes and platelets), CD3 (T cells), CD4 (T-cell subsets, also NKT cells, innate lymphoid cells and macrophages), CD8 (cytotoxic T cells), SLP76 (T-cell development and maturation marker), and FOXP3 (Treg cells) **(C)**. A representative image and quantitation of p-S6 staining (brown) in TLTs from wild-type and Atg7^−/−^ kidneys **(D)**. A representative image and quantitation of p-S6 in tubules in Atg7^−/−^ vs. wild-type mice **(E)**. Staining for p-S6 in tubules in close proximity to the TLTs (arrows) **(F)**. Fibrosis (red) was determined by second harmonic generation in the interstitium in +/+ and Atg7^−/−^ kidneys **(G)**. Student’s *t*-test was used for comparisons between two independent groups. A *p*-value of < 0.05 was considered statistically significant. Values are expressed as means ± SEM. Scale bar = 100 µm. *p < 0.05; ^**^ *p* < 0.01.

Next, IHC staining was performed in Atg7^−/−^ kidneys to characterize the presence of TLT markers. TLTs are characterized by PCNA, CD21, and C-X-C motif chemokine ligand 13 (CXCL13) staining and close proximity to blood vessels (1, 2). CXCL13 is a chemokine expressed by tissue fibroblasts that are thought to recruit B cells into the TLTs (2, 18). PCNA staining in tubules was increased in Atg7^−/−^ kidneys (**Figure 2A**). PCNA staining in tubules is a known response to tubular injury (46). There was PCNA (**Figure 2A**), CD21 (follicular dendritic cells), and CXCL13 (**Figure 2B**) staining in the TLTs (**Figure 2B**). Additional images of PCNA staining in TLTs in Atg7^−/−^ kidneys are demonstrated in **Supplementary Figure S1G**. Next, the cells present in the TLT were further characterized. There was staining in the TLTs for CD45 (a marker of all hematopoietic cells except for mature erythrocytes and platelets), CD3 (T cells), CD4 (helper T cells), CD8 (cytotoxic T cells), SLP-76 (T-cell development and maturation marker), and FOXP3 (Treg cells) (**Figure 2C**). CD3 is expressed by a high percentage of circulating peripheral T cells, forming a complex with the T-cell receptor (TCR). CD4 (cluster of differentiation 4) is a glycoprotein that serves as a co-receptor for the T-cell receptor (TCR) on CD4 T cells. Lymphocyte cytosolic protein 2, also known SLP-76, is an adaptor protein that plays a key role in signaling pathways within T cells and other immune cells (47). Studies using SLP-76-deficient T-cell lines or mice have provided strong evidence that SLP-76 plays a positive role in promoting T-cell development and activation (47).

As the proliferation of cells is a characteristic of T cells, p-S6, a downstream mTORC1 target and a potent mediator of proliferation, was studied. There was staining for p-S6 in TLTs in both +/+ and Atg7^−/−^ kidneys (**Figure 2D**), and p-S6 staining was increased in TLTs in Atg7^−/−^ compared to wild-type kidneys (**Figure 2D**). There was increased staining for p-S6 in tubules in Atg7^−/−^ versus wild-type mice (**Figure 2E**). There was staining for p-S6 in tubules in close proximity to the TLTs (**Figure 2F**).

Next, it was determined whether the presence of large TLTs in Atg^−/−^ kidneys was associated with increased fibrosis in the kidney. The percent fibrosis was low in the interstitium, and there was no difference in fibrosis between wild-type and Atg7^−/−^ kidneys (**Figure 2G**).

The cause of the proliferation of cells in TLTs is not well understood. mTORC1 is a well-known proliferative signaling pathway (17), and mTOR plays an important role in lymphocyte proliferation, differentiation, and survival (21). Immunoblot analysis for an extensive array of mTOR proteins in Atg7^−/−^ kidneys was performed (**Supplementary Figure S2**). The increase in p-S6^S240/244^ and p-Akt^T308^ (markers of mTORC1) but not pAkt^S473^, pSGK1^S422^, p-PKCα^T638/641^, p-GSK3β^S9^, or p-mTOR^S2481^ (markers of mTORC2) in Atg7^−/−^ kidneys suggested that mTORC1 but not mTORC2 was activated in the Atg7^−/−^ kidneys. There was no increase in p-ACC^S79^ (a marker of p-AMPK), phosphorylated eukaryotic translation initiation factor 4E-binding protein 1 (4E-BP1) isoforms, or p-c-Myc (a regulator of proliferation), in Atg7^−/−^ kidneys. The increase in p-S6, but no increase in p-4E-BP1 isoforms, suggests that mTORC1 signals downstream to p-S6 rather than 4E-BP1 in causing the proliferation of cells within the TLTs.

To throw light on early events in the tubules that could lead to the subsequent formation of TLTs, 180-day-old Atg7^−/−^ kidneys were studied. Small TLTs were seen in both wild-type and 180-old Atg7^−/−^ mice (**Supplementary Figures S3A–C**). The most prominent tubular response in these kidneys was a massive increase in p62 in the tubules surrounding the TLTs. There was intense staining for p62 in tubules in the whole kidney sections (**Supplementary Figures S3D, E**), cortex (**Supplementary Figures S3F, G**), and medulla (**Supplementary Figures S3H, I**). p62 is a marker of cell stress or damage (43, 45). Kidneys were also stained for VCAM-1, which is a well-known chemoattractant surrounding TLTs in ischemic kidneys (16). There was little VCAM-1 staining in the Atg7^−/−^ kidneys, that was not localized to tubules surrounding TLTs (**Supplementary Figures S3J–L**). TNF-α is the major cytokine seen in the TLTs in ischemic kidneys and is thought to play a role in promoting inflammation, recruiting immune cells, and organizing the tissue structure in TLTs (16). There was no TNF-α staining in the small TLTs seen in wild type or Atg7^−/−^ kidneys (**Supplementary Figures S3M, N**). There was increased tubular injury, as indicated by kidney injury marker-1 (KIM-1) staining, in Atg7^−/−^ kidneys (**Supplementary Figures S3O–Q**). In summary, at an early age in Atg7^−/−^ kidneys, there is tubular injury and p62 in the tubules was much more abundant than VCAM-1 and TNF-α. In conclusion, intense p62 staining, a marker of cell stress and tubular injury precedes TLT formation in Atg7^−/−^ kidneys.

### TLTs in Pkd1^RC/RC^ kidneys are virtually eliminated by the mTOR inhibitor Torin2

**Figure 3:**
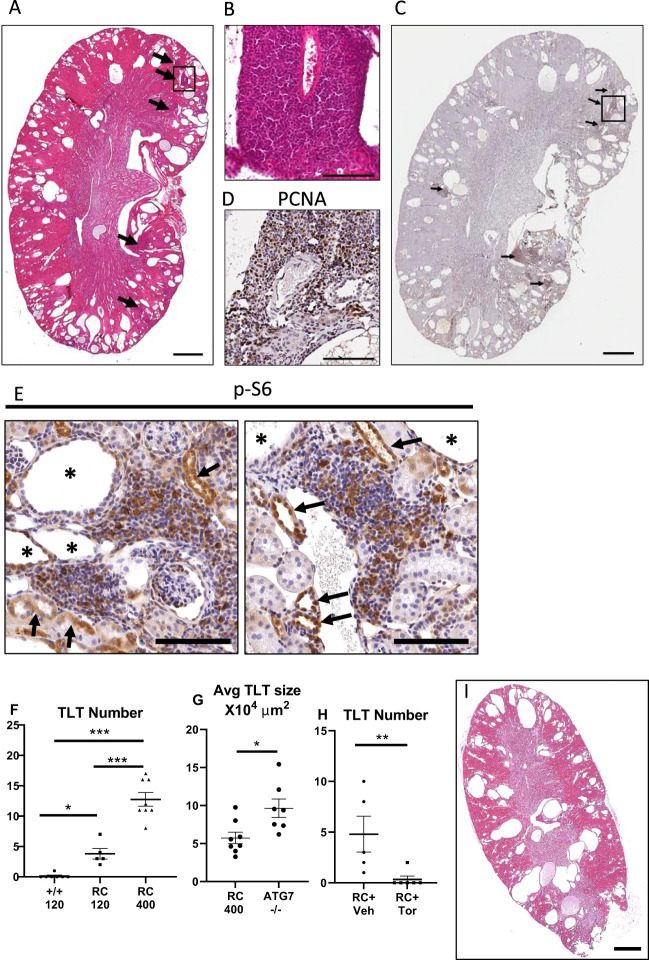
TLTs in Pkd1^RC/RC^ kidneys were virtually eliminated by the mTOR inhibitor Torin2. Representative images of hematoxylin–eosin **(A)** and PCNA (brown staining) **(C)** show TLTs (arrows) in Pkd1^RC/RC^ kidneys. Inserts show higher magnification of hematoxylin–eosin **(B)** and PCNA **(D)** in Pkd1^RC/RC^ kidneys. TLTs are shown surrounding blood vessels. p-S6 staining (brown) in tubules in close proximity to TLTs (arrows) and in the TLTs in Pkd1^RC/RC^ kidney **(E)**. Quantitation of the number and size (µm^2^) of TLTs was performed in midline longitudinal kidney sections **(F–H)**. Number of TLTs in wild-type, 120-day-old, and 400-day-old Pkd1^RC/RC^ kidneys **(F)**. Size of TLTs in 400-day-old Pkd1^RC/RC^ vs. 400-day-old Atg7^−/−^ kidneys **(G)**. Number of TLTs per kidney cross-section in Pkd1^RC/RC^ kidneys from mice treated with the mTOR inhibitor Torin2 (Tor) **(H)**. Hematoxylin–eosin image of a kidney from a Pkd1^RC/RC^ mouse treated with Torin2 shows the absence of TLTs **(I)**. Multiple group comparisons were performed using analysis of variance (ANOVA) with posttest according to the Newman–Keuls method. Student’s *t*-test was used for comparisons between two independent groups. A *p*-value of < 0.05 was considered statistically significant. Values are expressed as means ± SEM. Asterisk indicates cyst. ^*^ *p* < 0.05; ^***^ *p* < 0.001. Scale bar = 1,000 µm **(A, C, I)**. Scale bar = 100 µm **(B, D, E)**.

It is known that there is chronic interstitial inflammation with lymphocytes (25) and macrophages (48) in Pkd1^RC/RC^ mouse polycystic kidneys, a hypomorphic model of human autosomal dominant polycystic kidney disease (ADPKD). As chronic inflammation is known to play a role in the formation of TLTs (1), it was determined whether there were TLTs in the Pkd1^RC/RC^ mouse kidneys. TLTs were seen on low- and high-power magnification of hematoxylin–eosin-stained kidneys in 120-day-old Pkd1^RC/RC^ kidneys (**Figures 3A, B**; **Supplementary Figures S4A, B**). There was an intense proliferation of cells in the TLTs as indicated by staining for PCNA (**Figures 3C, D**; **Supplementary Figures S4C, D**).

Increased proliferation is a feature of cells lining cysts in PKD and also of the cells in the TLTs. There was also PCNA staining of the tubular cells lining the cysts (**Supplementary Figure S4D**), as we have previously described (26). Additional images of PCNA staining in TLTs in 120-day-old Pkd1^RC/RC^ kidneys are shown in **Supplementary Figure S4D**. There was increased p-S6 in the tubules in close proximity to TLTs in the TLTs in Pkd1^RC/RC^ kidneys (**Figure 3E**).

TLTs have been classified into three stages based on the presence of CD21-positive follicular dendritic cells (FDCs) and germinal centers (2). Stage 1 TLTs do not have CD21 FDCs. Stage 2 TLTs have CD21-positive cells but no germinal centers. TLTs with prominent CD21 FDCs and germinal centers are classified as stage 3. The TLTs in 120-day-old Pkd1^RC/RC^ kidneys had very slight CD21 staining and no germinal centers, suggesting that they were early-stage 1 TLTs (**Supplementary Figure S4E**). The chemokine CXCL13 is a strong factor for B-cell recruitment (2). There was very slight staining for CXCL13 in 120-day-old Pkd1^RC/RC^ kidneys (**Supplementary Figure S4F**).

The number of TLTs was increased in the kidney in 120-day-old and further increased in 400-day-old Pkd1^RC/RC^ mice compared to wild-type mice (**Figure 3F**). The size of TLTs in 400-day-old Pkd1^RC/RC^ mice was 50% smaller compared to the 400-day-old Atg7^−/−^ kidneys (**Figure 3G**). TLTs can show phenotypic heterogeneity, ranging from less organized structures composed of intermingled lymphocyte aggregates to highly organized structures made up of discrete clusters, sometimes even with germinal centers (1). The architecture of the TLTs in Pkd1^RC/RC^ kidneys (**Figure 3A**) was less organized, and the cells were less densely packed than the highly organized structures with densely packed cells seen in Atg7^−/−^ kidneys (**Figures 1B–D, F**). The less organized TLTs in Pkd1^RC/RC^ compared to Atg7^−/−^ kidneys may be due to the less advanced stage of the TLTs in Pkd1^RC/RC^ kidneys and the presence of large cysts, which results in the lack of interstitial space for TLTs to grow.

Increased proliferation (PCNA) (**Figures 3C, D**) and p-S6 (**Figure 3E**) in the TLTs was seen in Pkd1^RC/RC^ kidneys. The mTOR inhibitor Torin2 is a potent antiproliferative agent (26). Thus, the number and size of TLTs were determined in *Pkd1^RC^* ^/RC^ kidneys from mice treated with Torin2 at an early age, from days 50 to 120, using the dosing strategy we have previously described (26). The number of TLTs in *Pkd1* ^RC/RC^ kidneys was virtually eliminated by treatment with Torin2 (**Figures 3H, I**). In our published study, treatment of mice with Torin2 resulted in a 30% decrease in cyst index and improved kidney function (26).

### The number and size of TLTs in ischemic kidneys are significantly reduced by the mTOR inhibitor Torin2

**Figure 4:**
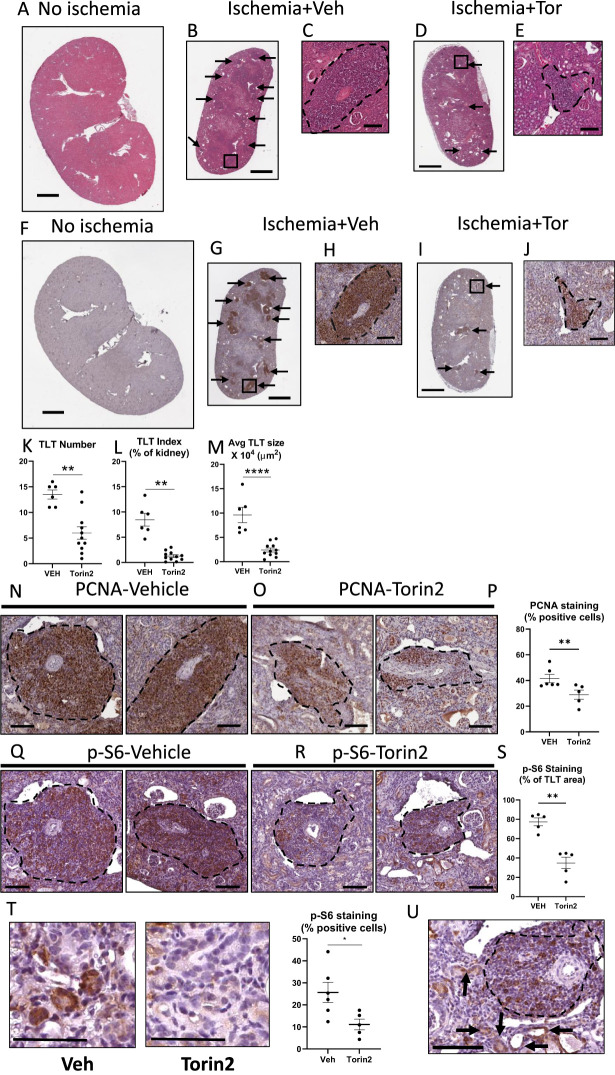
The number and size of TLTs, as well as PCNA and p-S6 staining in TLTs in ischemic kidneys, are significantly reduced by the mTOR inhibitor Torin2. TLTs (arrows) were observed at both low- and high-power magnification in hematoxylin–eosin-stained ischemic kidneys compared to no TLTs seen in the contralateral normal kidney **(A, B)**. Insert showing higher-power hematoxylin–eosin staining of TLT (square) in the ischemic kidney **(C)**. The presence of TLTs (arrows) in ischemic kidneys vs. no TLTs in contralateral normal kidneys was confirmed by staining for PCNA **(F, G)**. Insert showing higher-power PCNA staining of TLT (square) in the ischemic kidney **(H)**. Representative images of hematoxylin–eosin staining **(D)** and PCNA staining **(I)** in ischemic kidneys treated with Torin2 show significantly less TLT number, size, and index. Inserts showing higher-power hematoxylin–eosin **(E)** and PCNA staining of TLT **(J)** in the ischemic kidney. Quantitation of TLT number **(K)**, index **(L)**, and average size **(M)** in the vehicle and Torin2-treated mice. Staining for PCNA in the vehicle and Torin2-treated kidneys **(N, O)**. Percentage of PCNA-positive cells within the TLTs in the vehicle and Torin2-treated mice **(P)**. Staining for p-S6 in the vehicle and Torin2-treated kidneys **(Q, R)**. Percentage of the area within the TLTs staining for p-S6 in the vehicle and Torin2-treated mice **(S)**. Representative pictures of tubular staining for p-S6 and the percent of positive staining cells in Torin2 vs. vehicle-treated ischemic kidneys **(T)**. Staining for p-S6 in tubules in close proximity to the TLTs **(U)**. Student’s *t*-test was used for comparisons between two independent groups. A *p*-value of < 0.05 was considered statistically significant. Values are expressed as means ± SEM. Scale bar = 1,000 µm **(A, B, D, F, G, I)**. Scale bar = 100 µm **(C, E, H, J, N, O, Q, R, U)**. Scale bar = 50 µm **(T)**. ^*^ *p* < 0.05; ^**^ *p* < 0.01; ^****^ *p* < 0.0001. Three separate experiments were performed: (1) *N* = 3 vehicle, *N* = 5 Torin-treated, (2) *N* = 2 vehicle, *N* = 4 Torin-treated, and (3) *N* = 1 vehicle, *N* = 2 Torin-treated.

Based on the p-S6 staining seen in tubules and TLTs in +/+, Atg7^−/−^, and PKD kidneys, and studies that p-S6 is increased in tubules in ischemic kidneys in mice (49), we sought to examine whether TLTs were present in ischemic kidneys. Therefore, adult wild-type mice were subjected to 45 min of unilateral ischemia and the contralateral kidney was used as a control. Forty-five days after reperfusion, ischemic kidneys were examined for TLTs. After unilateral renal pedicle clamp in aged mice, the kidney phenotype shows an increase in kidney injury markers, inflammation fibrosis, and TLT formation and growth, which worsens with a longer period of renal pedicle clamp (30). TLTs were seen on low- and high-power magnification of hematoxylin-eosin-stained ischemic kidneys compared to no TLTs seen in the contralateral normal kidney (**Figures 4A–C**). Ischemic kidneys were smaller than the contralateral control kidney. There were TLTs with intense PCNA staining in ischemic kidneys versus no TLTs in contralateral normal kidneys (**Figures 4F–H**). Mice were treated with the mTOR inhibitor Torin2 for 35 days, starting from day 10 after the renal pedicle clamp until they were killed. Representative images of ischemic kidneys treated with Torin2 showing significantly less TLT number, size, and index are shown in **Figures 4D, E, I, J**. The number, TLT index, and size of TLTs were significantly reduced by treating ischemic acute kidney injury (AKI) mice with the mTOR inhibitor Torin2 (**Figures 4K–M**). The percentage of cells within the TLTs staining for PCNA was significantly reduced by treatment with Torin2 (**Figures 4N–P**), indicating that Torin2 reduces the proliferation of cells in the TLTs. TLTs in ischemic kidneys showed intense staining for p-S6 (**Figure 4Q**). Staining for p-S6 and size of TLTs was significantly reduced by treatment of mice with Torin2 (**Figure 4R**). The percent of TLT staining with p-S6 was significantly less in Torin2 versus vehicle-treated ischemic kidneys (**Supplementary Figure S4**). Additional images of TLTs, PCNA, and p-S6 staining in vehicle versus Torin2-treated ischemic kidneys are shown in **Supplementary Figures S5A–L**. There was staining for p-S6 in tubules in ischemic kidneys that was reduced by Torin2 (**Figure 4T**). There was staining for p-S6 in tubules in close proximity to the TLTs (**Figure 4U**).

### The mTOR inhibitor Torin2 reduces CD3, CD20, CD21, and p62 staining in TLTs in ischemic kidneys

**Figure 5:**
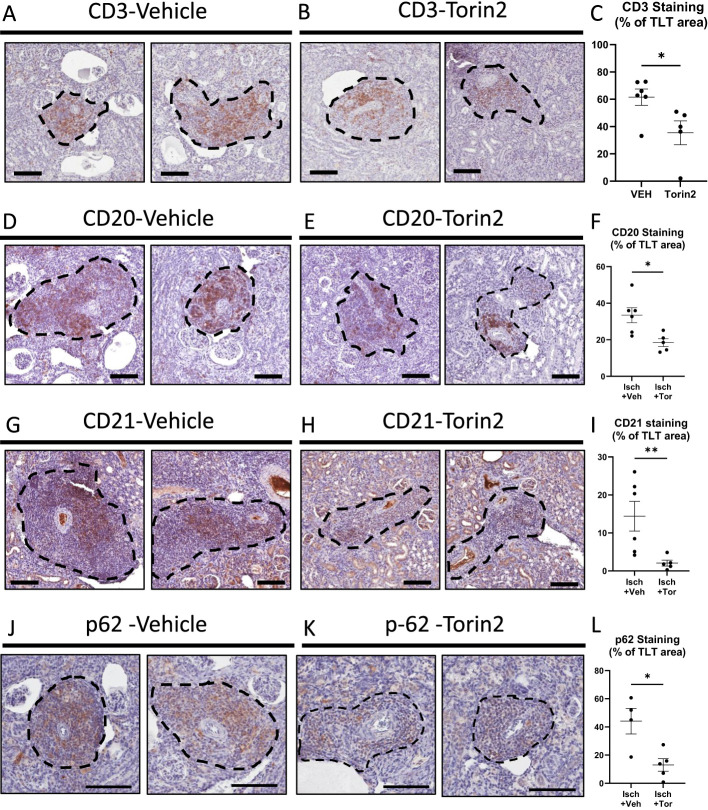
The mTOR inhibitor Torin2 reduces CD3, CD20, and CD21 staining in TLTs in ischemic kidneys. Staining for CD3, a T-cell marker **(A)**, CD20, a B-cell marker **(D)**, and CD21, a marker of follicular dendritic cells (FDCs) **(G)** in TLTs in the vehicle and Torin2-treated mice. The effect of Torin2 on staining with CD3 **(B)**, CD20 **(E)**, and CD21 **(H)**. Quantitation of CD3 **(C)**, CD20 **(F)**, and CD21 **(I)** staining in the vehicle and Torin2-treated mice. Quantitation of p62 in TLTs in vehicle and Torin2-treated mice **(J–L)**. Student’s *t*-test was used for comparisons between two independent groups. A *p*-value of < 0.05 was considered statistically significant. Values are expressed as means ± SEM. ^*^ *p* < 0.05; ^**^ *p* < 0.01. Scale bar = 100 µm.

Torin2 resulted in a decrease in staining for CD3, a marker of T cells; CD20, a B-cell marker; and CD21, a marker of FDCs, in TLTs in ischemic kidneys (**Figures 5A–I**). There was a decrease in staining (% of TLT area) for CD3, CD20, and CD21 in Torin2-treated mice (**Figures 5C, F, I**). Torin2 resulted in a nearly complete inhibition of CD21 staining in the TLTs. T and B cells and FDCs are three of the major components of TLTs. CD21 follicular dendritic cells organize B-cell homeostasis and humeral responses in TLTs and are essential for B-cell function in TLTs (2).

There was intense p62 staining, a marker of suppressed autophagy, in immune cells in TLTs in vehicle-treated ischemic kidneys (**Figures 5J, L**). p62 staining was reduced, indicating increased autophagy in Torin2-treated ischemic kidneys (**Figures 5K, L**).

### Minimal staining for p-AKT^S473^ (a marker of mTORC2) in TLTs

Torin2 has been described to inhibit mTORC2 (26), so p-AKT^S473^, a marker of mTORC2 activation, was measured in the TLTs. The best-characterized target of mTORC2 is the AKT kinase, and mTORC2 is known to phosphorylate AKT on Serine473 (50). TLTs in aged wild-type, Atg7^−/−^, Pkd1^RC/RC^, and ischemic kidneys were stained for p-AKT^S473^. Sections from lung cancer tumors from our previous studies (51) were used as a positive control and showed intense staining for p-AKT^S473^ (**Supplementary Figure S6A**). There was minimal staining for p-AKT^S473^ in a few of the TLTs in aged wild-type (**Supplementary Figure S6B**) and aged Atg7^−/−^ (**Supplementary Figure S6C**) kidneys. There was no staining for p-AKT^S473^ in any of the TLTs in Pkd1^RC/RC^ (**Supplementary Figure S6D**) or ischemia (**Supplementary Figure S6E**) kidneys. This is compared to the intense staining for p-S6, a marker of mTORC1, seen in all the TLTs from aged wild type, Atg7^−/−^, Pkd1^RC/RC^, and ischemic kidneys. Thus, it is unlikely that the effect of Torin2 on TLTs was related to inhibition of mTORC2.

### Tubule-specific Raptor knockout has no significant effect on TLTs

A common feature of Atg7^−/−^ kidneys, polycystic kidneys, and ischemic kidneys was p-S6 staining in the tubules in close proximity to TLTs. The mTOR inhibitor Torin2 resulted in a decrease of p-S6 in both the tubules and TLTs in ischemic kidneys (**Figures 4Q–T**). To determine whether p-S6 in tubules versus TLTs was driving proliferation, formation, and growth in TLTs, tubule-specific Atg7^−/−^Raptor^−/−^ mice and Atg7^−/−^ controls were exposed to ischemia to induce TLT formation. Raptor, the functional unit of mTORC1, was knocked out in the tubules in Atg7^−/−^ mice. Renal tubule-specific cadherin Cre Raptor^−/−^ mice (KSP1.3 Cad Cre Raptor^−/−^ mice) were developed by Cre recombinase recombination. The hypothesis was that if increased p-S6 in the tubules was driving TLT formation and growth, the Atg7^−/−^Raptor^−/−^ mice exposed to ischemia would have fewer TLTs than control Atg7^−/−^ mice exposed to ischemia.

**Figure 6:**
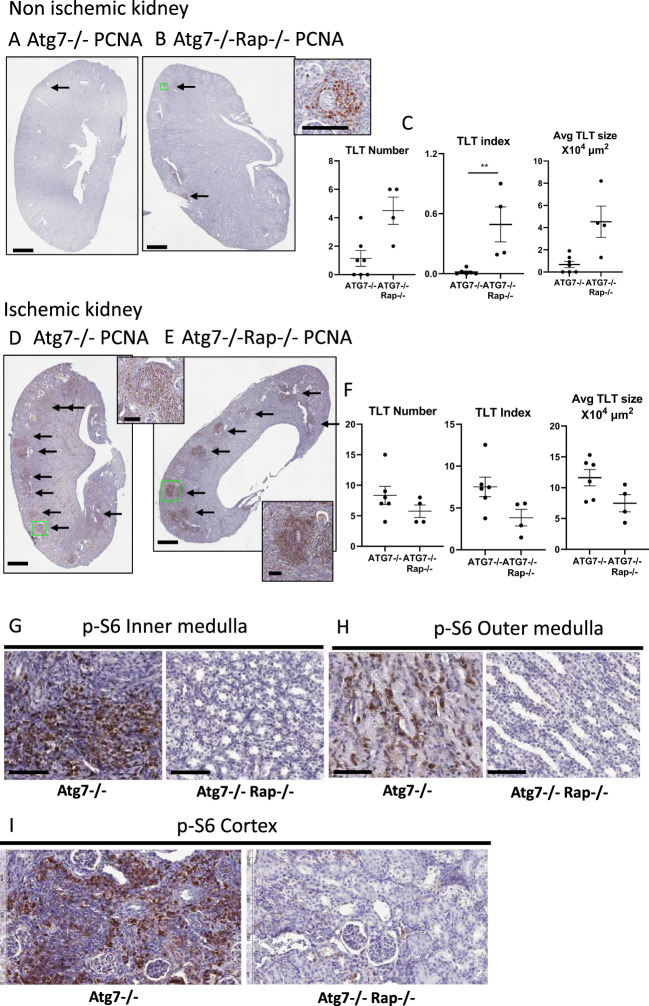
Tubule-specific Raptor (mTORC1) knockout has a small effect on TLTs in ischemic but no effect on TLTs in nonischemic kidneys. Representative PCNA-stained sections in **(A)** Atg7^−/−^ kidney and **(B)** Atg7^−/−^Raptor^−/−^ kidneys. Inset shows higher magnification of a small TLT in an Atg7^−/−^Raptor^−/−^ kidney. The number of TLTs (arrows) and TLT index (percent of kidney cross-section) in contralateral control nonischemic kidneys **(C)**. Representative PCNA-stained sections in **(D)** Atg7^−/−^ kidney and **(E)** Atg7^−/−^Raptor^−/−^ kidneys. The number of TLTs (arrows) and TLT index (percent of kidney cross-section) in ischemic kidneys **(F)**. Inserts show a higher magnification of TLTs. p-S6 staining in **(G)** inner medulla, **(H)** outer medulla, and **(I)** cortex in Atg7^−/−^ and Atg7^−/−^Raptor^−/−^ kidneys. Student’s *t*-test was used for comparisons between two independent groups. A *p*-value of <0.05 was considered statistically significant. Values are expressed as means ± SEM. ^**^ *p* < 0.01. Scale bar = 1 mm **(A, B, D, E)**. Scale bar = 100 µm [inserts, **(C, F–I)**]. Rap = Raptor. 2 Separate experiments were performed: (1) *N* = 3 Atg7^−/−^, *N*=3 Atg7^−/−^, Raptor^−/−^, 2) *N* = 3 Atg7^−/−^, *N* = 1 Atg7^−/−^, Raptor^−/−^.

In PCNA-stained sections of contralateral control nonischemic kidneys, the number of TLTs was not significantly different in Atg7^−/−^ (**Figure 6A**) vs. Atg7^−/−^Raptor^−/−^ kidneys (**Figure 6B**). The TLT index was higher, rather than lower, in Atg7^−/−^Raptor^−/−^ kidneys (**Figure 6B**). However, TLTs were very small, as indicated by a very low TLT index, as shown in the higher magnification insert of **Figure 6B**. The quantitation of TLT number and index is shown in **Figure 6C**.

In PCNA-stained sections of ischemic kidneys, the number of TLTs was the same in Atg7^−/−^ (**Figure 6D**) vs. Atg7^−/−^Raptor^−/−^ kidneys (**Figure 6E**), and the TLT index was slightly lower in Atg7^−/−^Raptor^−/−^ kidneys (**Figure 6E**). High magnification inserts of TLTs in Atg7^−/−^ (**Figure 6D**) and Atg7^−/−^Raptor^−/−^ kidneys (**Figure 6E**) are shown. The quantitation of TLT number and index is shown in **Figure 6F**.

There was decreased p-S6 staining in tubules in the inner and outer medulla, as well as in the cortex, in Atg7^−/−^ and Rictor^−/−^ mice (**Figures 6G–I**).

In summary, there was p-S6 staining in tubules in close proximity to TLTs. However, knockout of mTORC1 in tubules reduces p-S6 staining in the cortex and medulla but does not affect the number of TLTs.

### Torin2 reduces TLTs in *Pkd1* ^RC/RC^ and ischemic kidneys independently of an effect on tubular injury

Kidneys exposed to ischemia develop tubular injury, and tubular injury can be a stimulus for TLT formation and growth (2). Therefore, the relationship between tubular injury (KIM-1 staining) and TLTs was investigated in Atg7^−/−^, *Pkd1* ^RC/RC^, and ischemic kidneys. KIM-1 is a marker of kidney tubular injury and regeneration in mice (51) and in humans (52).

**Figure 7:**
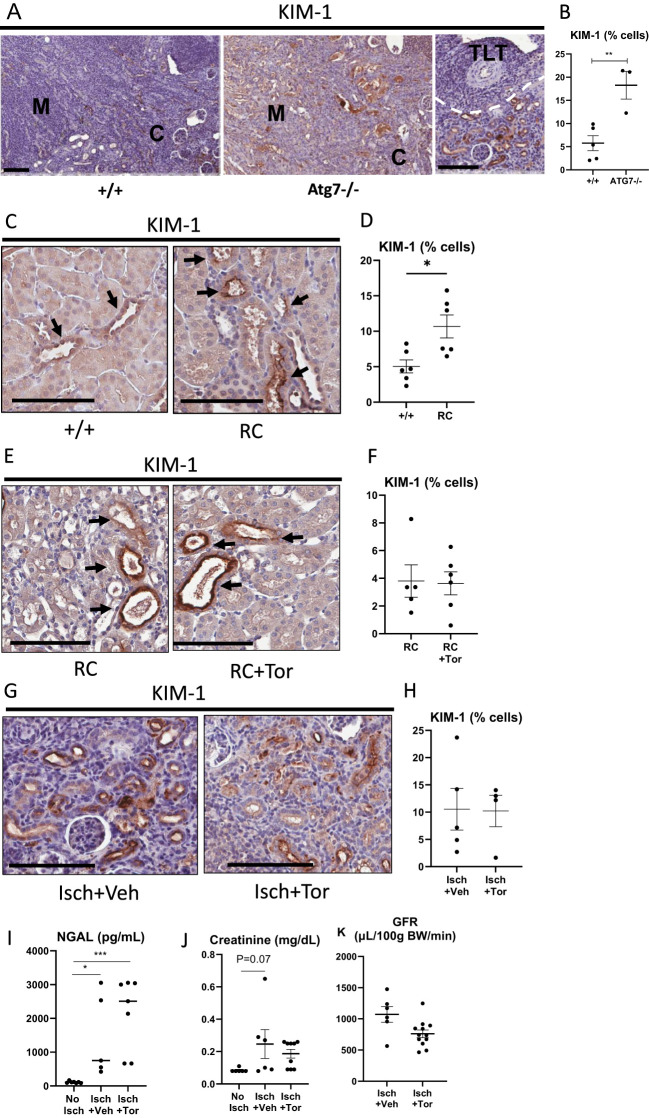
Increased tubular injury in Atg7^−/−^, *Pkd1* ^RC/RC^, and ischemic kidneys. The mTOR inhibitor Torin2 has no effect on tubular injury. KIM-1 staining (brown) in cortex **(C)** and medulla **(M)** in +/+ and Atg7^−/−^ kidneys **(A)**. KIM-1 staining in tubules in close proximity to a TLT **(A)**. Quantitation of KIM-1 staining **(B)**. Kidney injury marker-1 (KIM-1) staining was determined in the outer stripe of the outer medulla in ischemic kidneys and *Pkd1* ^RC/RC^ kidneys. KIM-1 staining (brown) in +/+ and *Pkd1* ^RC/RC^ (RC) kidneys **(C)**. Quantitation of KIM-1 staining in +/+ and RC kidneys **(D)**. KIM-1 staining in a separate experiment in RC and RC mice treated with Torin2 **(E)**. Quantitation of KIM-1 staining in RC and RC +Torin2 kidneys **(F)**. KIM-1 staining (brown) in ischemic (Isch) kidneys in mice treated with vehicle (Veh) or Torin2 (Tor) **(G)**. Quantitation of KIM-1 staining in kidneys in mice treated with vehicle (Veh) or Torin2 (Tor) **(H)**. Serum NGAL from sham-operated mice (no ischemia), ischemic kidney in mice treated with vehicle, and ischemic kidney from mice treated with Torin2 **(I)**. Serum creatinine from sham-operated mice (no ischemia), ischemic kidney in mice treated with vehicle, and ischemic kidney from mice treated with Torin2 **(J)**. Transcutaneous glomerular filtration rate (GFR) measurements in ischemic (Isch) kidneys in mice treated with vehicle (Veh) or Torin2 (Tor) **(K)**. Student’s *t*-test was used for comparisons between two independent groups. A *p*-value of < 0.05 was considered statistically significant. Values are expressed as means ± SEM. ^*^ *p* < 0.05; ^**^ *p* < 0.01; ^***^ *p* < 0.001. Scale bar = 100 µm. BW, body weight.

KIM-1 staining in tubules was increased in Atg7^−/−^ vs. age-matched wild-type mice, indicating tubular injury in Atg7^−/−^ tubules (**Figure 7A**). Representative images of KIM-1 staining and KIM-1 staining in tubules close to a TLT (**Figure 7A**) and quantitation of KIM-1 staining (**Figure 7B**) are shown. As tubular injury is known to produce cytokines and chemokines that play a role in TLT formation and growth (16, 53), an array of proinflammatory cytokines was measured in Atg7^−/−^ kidneys (**Table 1**). There was an increase in tumor necrosis factor-α (TNF-α), IL-1β, IL-2, IL-6, and C-X-C motif chemokine ligand 1 (CXCL1) in Atg7^−/−^ vs. age-matched wild-type control kidneys (**Table 1**). As TLTs form in on average less than 4% of the kidney and tubules make up the bulk of the kidney, and injured kidney tubules are known to produce TNF-α, IL-1β, IL-6, and CXCL1 (54), it is likely that the major source of such large increases in cytokines was coming from the injured tubules rather than the TLTs. Proinflammatory cytokines like TNF-α, IL-1β, IL-2, IL-6, and CXCL1 are known to play a role in TLT formation and growth (16, 53), so it is likely that these cytokines produced by the tubules surrounding the TLTs were contributing to TLT formation and growth in Atg7^−/−^ kidneys.

**Table 1:** Proinflammatory cytokines in the kidney in wild-type (+) mice vs. Atg7^−/−^ mice.

	+/+ (*N* = 5)	Atg7^−/−^ (*N* = 5)
IFN-γ	0.04 ± 0.1	0.07 ± 0.2
TNF-α	4.9 ± 1.0	9.7 ± 1.4^*^
IL-1β	43.0 ± 16.5	129.4 ± 49.9^#^
IL-2	1.0 ± 0.3	4.9 ± 1.7^#^
IL-5	1.1 ± 0.4	1.5 ± 0.6
IL-6	19.6 ± 6.7	91.7 ± 42.9^##^
CXCL1	15.0 ± 5.2	78.0 ± 30.9^*^
IL-10	5.0 ± 2.0	5.7 ± 0.9
IL-12	28.3 ± 11.2	54.0 ± 24.8

Unit: pg/mL. Values are expressed as the mean ± SEM. ^*^ *p* < 0.05; ^#^ *p* = 0.05; ^##^ *p* = 0.09.

Next, the relationship between tubular injury and TLTs was investigated in *Pkd1* ^RC/RC^ kidneys. KIM-1 staining was increased in normal tubules in *Pkd1* ^RC/RC^ mice versus wild-type controls (**Figure 7C**). Representative images of KIM-1 staining (**Figure 7C**) and quantitation of KIM-1 staining (**Figure 7D**) are shown. In a separate analysis of IHC, Torin2 that reduced TLTs had no effect on KIM-1 staining (**Figures 7E, F**). These data suggest that Torin2 reduces TLTs in *Pkd1* ^RC/RC^ kidneys independently of an effect on tubular injury.

The outer stripe of the outer medulla (S3 segment of proximal tubules) is considered to be the major site of tubular injury in renal ischemia (55, 56). In the renal ischemia experiments, KIM-1 was quantitated in the outer stripe of the outer medulla. In ischemic kidneys, there was large KIM-1 staining in tubules, as expected. Torin2 did not affect KIM-1 staining in ischemic kidneys. Representative images of KIM-1 staining (**Figure 7G**) and quantitation of KIM-1 staining (**Figure 7H**) are shown. As expected, serum NGAL, a marker of kidney injury (51, 52), and serum creatinine were increased in mice with kidney ischemia compared to a group of age-matched mice without kidney ischemia (**Figures 7I, J**). There was no difference in serum NGAL or serum creatinine between vehicle- or Torin2-treated mice with kidney ischemia (**Figures 7I, J**). Torin2 did not have a significant effect on kidney function as determined by transcutaneous glomerular filtration rate (GFR) measurements (**Figure 7K**). There was a significant decrease in ATN scores in ischemic kidneys treated with Torin2 (**Supplementary Figures S7A–E**). There was minimal fibrosis in contralateral nonischemic kidneys (**Supplementary Figures S7F, H, J**). Torin2 reduced TLTs but did not affect fibrosis in ischemic kidneys (**Supplementary Figures S7G, I, J**). In summary, the reduction of TLTs was associated with less tubular necrosis but no effect on KIM-1 (a marker of tubular injury and regeneration) or kidney function. Interstitial fibrosis, a known consequence of chronic tubular injury (57), was not affected by Torin2.

### Tubule–TLT interactions

**Figure 8:**
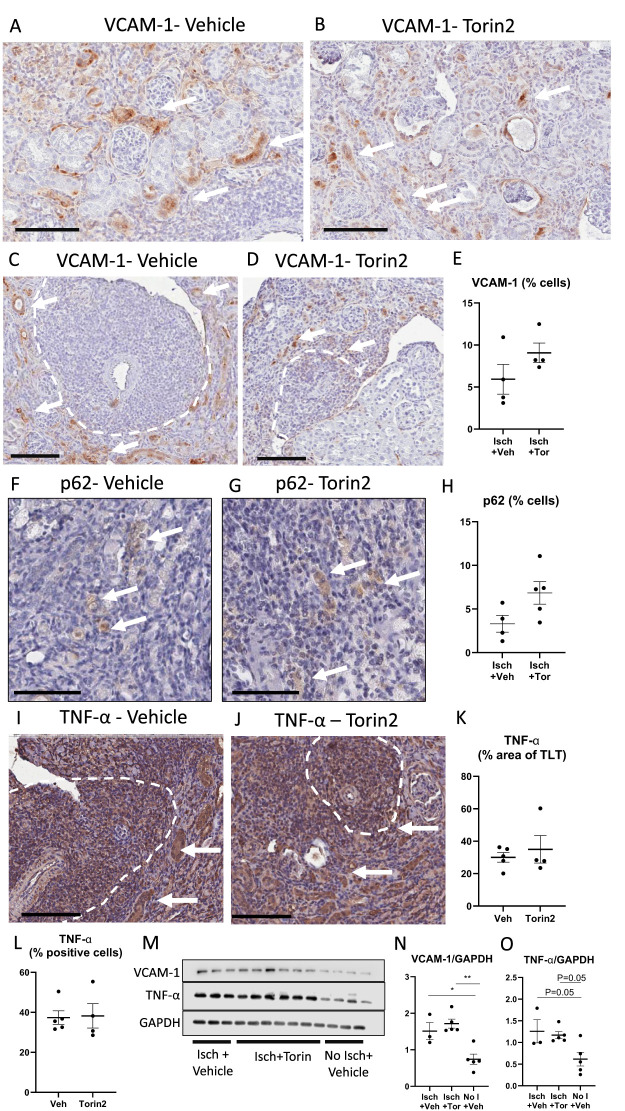
Torin2 did not have a significant effect on VCAM-1, p62, and TNF-α in ischemic kidneys. VCAM-1 staining in the tubules distant from TLTs **(A, B)** and in tubules surrounding TLTs **(C, D)** in both vehicle and Torin2-treated ischemic kidneys. Quantitation of VCAM-1 staining **(E)**. VCAM-1 staining (brown) is shown by arrows. p62 staining in the vehicle and Torin2-treated kidneys **(F, G)**. Quantitation of p62 staining **(H)**. p62 staining (brown) is shown by arrows. TNF-α staining in TLTs and tubules surrounding TLTs in the vehicle and Torin2-treated kidneys **(I, J)**. Quantitation of TNF-α staining in TLTs **(K)** and in the tubules **(L)**. TNF-α staining (brown) is shown by arrows. TLTs are outlined by dashed lines. The effect of ischemia and Torin 2 on VCAM-1 and TNF-α in immunoblot analysis for whole kidney homogenates **(M)**. Densitometric analysis of VCAM-1 **(N)** and TNF-α **(O)**. Isch, ischemia. Scale bar = 100 µm. ^*^ *p* < 0.05; ^**^ *p* < 0.01.

Next, VCAM-1, p62, and TNF-α were explored as potential tubule-derived factors surrounding TLTs that may be influenced by mTOR. VCAM-1 is the main chemokine in the tubules surrounding TLTs and is thought to play a role in TLT development (16). There was VCAM-1 staining in the tubules distant from TLTs (**Figures 8A, B**) and in tubules surrounding TLTs (**Figures 8C, D**) in both vehicles and Torin2-treated ischemic kidneys. Torin2 did not have a significant effect on VCAM-1 staining (**Figure 8E**). p62 was prominent in tubules around TLTs in Atg7^−/−^ mice (**Figure 1F**), so p62 in tubules in ischemic kidneys was studied. p62 plays a role in inflammatory responses, like inflammasome activation (43), expression of inflammatory genes via NF-κB (43), and production of chemokines like CXCL1 that could potentially influence TLT formation and growth (58). p62 was seen in a few tubules in both vehicle- and Torin2-treated kidneys (**Figures 8F, G**). p-62 was not seen in the tubules surrounding TLTs in ischemic kidneys. Torin2 treatment had no significant effect on p62 in tubules (**Figure 8H**). TNF-α is a major pro-inflammatory cytokine known to be expressed in injured tubules (59) and TLTs (16) in ischemic kidneys. TNF-α was seen in tubules surrounding TLTs and in TLTs (**Figures 8I, J**), and Torin2 had no effect on TNF-α in tubules or in TLTs (**Figures 8K, L**). Next, the effect of ischemia and Torin2 on VCAM-1 and TNF-α in immunoblot analysis of whole kidney homogenates was determined. Ischemia resulted in an increase in VCAM-1 and TNF-α compared to nonischemic kidneys (**Figures 8M–O**). Torin2 had no effect on VCAM-1 and TNF-α in ischemic kidneys (**Figures 8M–O**). In summary, these data suggest that mTOR-mediated tubule-TLT interactions in ischemic kidneys are independent of VCAM-1, p62, and TNF-α in the tubules.

## Discussion

The present study describes the following novel findings: (1) numerous, large, and discrete TLTs in aged tubule-specific Atg7^−/−^ kidneys; (2) intense p-S6 staining in TLTs in both wild-type and Atg7^−/−^ kidneys; (3) p-S6 staining in TLTs in PKD kidneys and virtual elimination of TLTs following treatment with Torin2, a potent mTOR inhibitor; (4) p-S6 staining in TLTs in ischemic kidneys and a significant reduction in both p-S6 staining and the number and size of TLTs by mTOR inhibition. Torin2 resulted in a decrease in the number as well as the size of TLTs, suggesting that it reduced both the formation and growth of TLTs; (5) a decrease in proliferation of cells in TLTs and reduced staining for the major components of TLTs—CD3 (T cells), CD20 (B cells), and CD21 (follicular dendritic cells)—in ischemic kidneys with mTOR inhibition; and (6) suppressed autophagy and increased proliferation in the cells in TLT-residing cells, both of which were reversed by mTOR inhibition. It is not well known what drives the proliferation of cells in TLTs, and these data provide new information that mTOR drives this proliferation process. This study has important therapeutic implications, as TLTs are present in many disease processes in multiple organs, and mTOR inhibitors are used in clinical practice.

The structural characteristics of TLTs have recently been described (2). The main characteristic that defines TLTs and separates TLTs from simple lymphocytic infiltration is organized lymphocytic aggregates with signs of proliferation, often in close proximity to blood vessels, sometimes completely surrounding a blood vessel. Other characteristics of TLTs depend on the stage of TLT formation and the underlying disease process. TLTs stain positive for CXCL13, a driving force for B-cell recruitment that is being made by CXCL13-producing fibroblasts (18). The presence of FDCs, as indicated by positive CD21 staining, is usually seen at a later stage of TLT formation. FDCs act as antigen-presenting cells within the TLTs, allowing interactions between cells in the TLTs (1). The TLTs in the aged Atg7^−/−^ kidneys showed all of the above characteristics.

We were intrigued by the large amount of p-S6 seen in the TLTs and surrounding tubules in Atg7^−/−^ kidneys, so next we determined the role of mTOR in TLT development and growth in two separate models of kidney disease, ADPKD and renal ischemia.

ADPKD is the most common hereditary kidney disease and is characterized by cyst formation and kidney failure. Based on the presence of chronic interstitial inflammation in Pkd1^RC/RC^ mouse kidneys (25, 48) and in human PKD kidneys (60) and that chronic inflammation is known to play a role in the formation of TLTs (1), Pkd1^RC/RC^ kidneys were examined for the presence of TLTs. There was intense p-S6 staining in the TLTs and surrounding tubules in Pkd1^RC/RC^ kidneys. TLTs were virtually eliminated in kidneys from our previous study of Pkd1^RC/RC^ mice treated with Torin2 (26), a potent mTOR inhibitor (27), suggesting that mTOR drives both the formation and growth of TLTs. The presence of TLTs, p-S6 staining in TLTs, and the role of mTOR in the formation and growth of TLTs have not previously been described in any organ system, specifically in PKD kidneys.

Ischemic AKI is a common problem in hospitalized patients, and ischemic AKI in the intensive care unit carries a high mortality. In a recent study, single-nucleus RNA sequencing (snRNA-seq) with results validated by immunostaining, *in situ* hybridization, and *in vitro* studies was performed on ischemic kidneys with TLTs in a mouse model similar to that used in the present study (16). snRNA-seq showed proinflammatory and profibrotic VCAM1+ injured proximal tubules with NF-κB and IFN-inducible transcription factor activation. On immunostaining, VCAM1+ tubules were preferentially localized around TLTs. Lymphocytes in TLTs expressed high levels of TNF-α and IFN-γ. Proinflammatory fibroblasts showed increased chemokine or cytokine production, thought to be contributing to lymphocyte recruitment and survival. TLTs and surrounding tubular cells showed an inflammatory phenotype via the production of multiple cytokines and chemokines (16). It was concluded that TLTs potentially amplify inflammation by providing a microenvironment that allows intense interactions between renal tubular and immune cells (16). Based on the prominent role of VCAM-1 and TNF in this gene profiling study and the intense p62 staining surrounding TLTs in Atg7^−/−^ kidneys, VCAM-1, TNF-α, and p62 were explored as potential tubule-derived factors surrounding TLTs that may be influenced by mTOR. Torin2 had no effect on VCAM-1 and p62 in tubules or TNF-α in tubules and TLTs.

p-S6 staining was seen in both TLTs and surrounding tubules in Atg7^−/−^ mice, *Pkd1* ^RC/RC^ mice, and ischemic kidneys. To determine the mechanistic role of mTORC1 in the tubules in TLT formation and growth, Raptor, which is essential for mTORC1 activity (61), was knocked out in the tubules in Atg7^−/−^ mice. In Raptor^−/−^ Atg7^−/−^ mice, there was a significant decrease in p-S6 staining in the tubules of both the cortex and medulla. However, there was no significant decrease in the number or size of TLTs in either nonischemic or ischemic kidneys. On the other hand, the mTOR inhibitor Torin2 which inhibited p-S6 in tubules and in TLTs, resulted in a large decrease in TLT number and size in *Pkd1* ^RC/RC^ and ischemic kidneys. These data suggested that p-S6 activity within the TLTs and not p-S6 activity in tubules is required for the proliferation of immune cells in TLTs and TLT formation and growth.

Next, the relationship between tubular injury and mTORC1-driven TLT formation and growth in Atg7^−/−^ kidneys was considered. Tubular injury is a known stimulus for TLT formation and growth (1). The presence of KIM-1 staining in tubules is a sensitive marker of tubular injury (51, 52). In Atg7^−/−^ kidneys, there was KIM-1 staining in tubules in close proximity to TLTs and a large increase in proinflammatory cytokines TNF-α, IL-1β, IL-6, and CXCL1, which are known to increase TLT formation and growth (53). A consistent finding in both early and late Atg7^−/−^ kidneys was intense p62 staining in tubules surrounding the TLTs and tubular injury. p62 is a marker of cell stress or damage, and its accumulation indicates a buildup of damaged proteins and cellular debris due to impaired autophagy (43, 45). High levels of p62 suggest the cell is experiencing stress and struggling to properly degrade damaged components (45). Thus, it is likely that tubular injury, increased p62 in tubules, and the resultant intense proinflammatory phenotype contributed to TLT formation and growth in Atg7^−/−^ mice. However, in kidney ischemia, Torin2 significantly reduced TLTs and decreased tubular necrosis without an effect on KIM-1. KIM-1 is expressed on surviving dedifferentiated renal proximal tubule epithelial cells undergoing regeneration after ischemic injury rather than being expressed on necrotic cells themselves (64). These data suggest that mTOR inhibition has the potential to reduce TLTs and tubular necrosis but does not improve tubular cell recovery and regeneration. The known essential role of mTORC1 in tubular homeostasis and tubular recovery and regeneration (62, 63) may explain why mTOR inhibition could reduce tubular necrosis without an effect on KIM-1, a marker of tubular regeneration and recovery.

**Figure 9:**
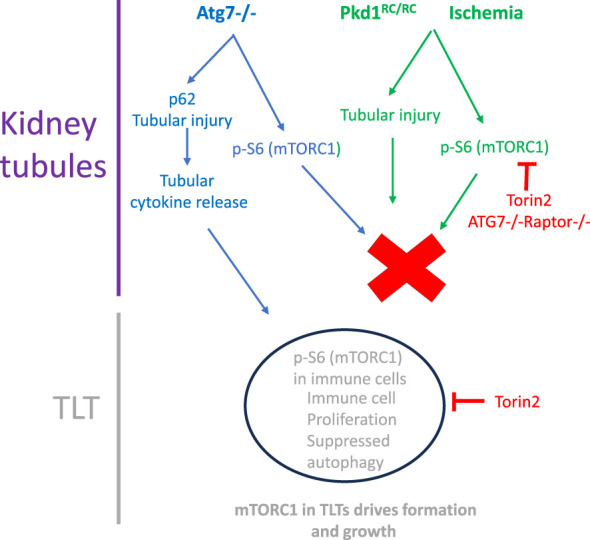
Schematic of how mTORC1 drives TLT growth. p-S6 staining is observed in tubules surrounding TLTs and in TLTs in Atg7^−/−^, Pkd1^RC/RC^ and ischemic kidneys. The mTOR inhibitor Torin2 reduces p-S6 staining in both tubules and TLTs. Knockout of mTORC1 (Raptor) in the tubules in Atg7^−/−^ mice had little effect on TLT formation and growth demonstrating that mTOR activity within the TLTs and not mTOR activity in tubules is required for p-S6-mediated proliferation of immune cells in TLTs and TLT formation and growth. There is a tubular injury in tubules surrounding TLTs in Atg7^−/−^, *Pkd1* ^RC/RC^, and ischemic kidneys. Torin2 decreased the number of TLTs in *Pkd1* ^RC/RC^ and ischemic kidneys but had no effect on tubular injury. There is suppressed autophagy of cells in TLTs. Torin2 increased autophagy, decreased proliferation, and decreased TLT formation/growth. X, no effect.

Next, the mechanism of how mTOR in the TLTs drives TLT formation and growth was considered. TLT formation and growth depend on T and B lymphocytes and CD21 FDCs, which are the major components of TLTs. mTOR inhibitors target lymphocyte proliferation and function (19). mTOR controls cell cycle progression in T lymphocytes (65), B-lymphocyte development and function (20), and regulates dendritic cell differentiation and function (66). In the present study, mTOR inhibition resulted in decreased CD3, a marker of T cells; CD20, a marker of B cells; and CD21, a driver of B-cell function in TLTs (2). Thus, it is likely that mTOR-driven proliferation of T cells, B cells, and FDCs, the major immune cells in TLTs, results in TLT formation and growth. It was also determined whether proliferation was tied to the autophagy of immune cells in TLTs. In ischemic kidneys, there was intense p62 staining in immune cells in TLTs, indicating suppressed autophagy. Suppressed autophagy is known to increase proliferation by postulated mechanisms involving decreased cellular senescence or limiting cell cycle progression (67). Torin2 resulted in reduced p62 staining, indicating increased autophagy in the immune cells. These data demonstrate that one of the mechanisms by which mTOR may promote TLT formation and growth is by suppressing autophagy in immune cells in TLTs, resulting in increased proliferation and TLT growth. A schematic of how the mTOR milieu in both tubules and TLTs fits into the proliferation of immune cells in TLTs and TLT formation and growth of TLTs is shown in **Figure 9**.

There are other potential signaling pathways that could drive the proliferation of immune cells in TLTs. The JAK-STAT pathway can mediate the proliferation of immune cells by activating the transcription of genes that promote cytokine and growth factor-mediated cell division and proliferation (68). Proinflammatory fibroblasts have STAT1 activation, producing chemokines and cytokines, including CXCL9/10 and B-cell-activating factor in TLTs (16). Wnt/β-catenin signaling stimulates pathways that promote cell cycle progression in immune cells, especially in the tumor microenvironment (69, 70). In aging mouse kidneys, there are lymphocytes in TLTs that express components of the Wnt-signaling pathway (71). c-Myc causes immune cells to proliferate by acting as a transcription factor that directly regulates the expression of numerous genes involved in cell cycle progression (72). The mechanistic role of the STAT1 and Wnt pathways in TLT formation and growth has not been studied. The present study showed the mechanistic role of mTOR signaling in the formation and growth of TLTs. We focused on the mTORC1 pathway as it mediates multiple cellular pathways in many different tissues, is highly conserved across species, responds to multiple stimuli like growth factors, hormones, and stress stimuli, and because of its intricate link to autophagy knockout, PKD, and AKI, the focus of the present study.

The specific common process that drives TLT formation and growth in general is not known. The present study suggests the role of mTOR in the proliferation of cells in TLTs in general, as intense p-S6 staining in TLTs was seen in aged wild-type kidneys and in three different kidney models: aged kidney-specific Atg7^−/−^ mice, PKD, and renal ischemia. As proliferation of lymphocytes is universal to all TLTs, and mTORC1 is well known to cause proliferation of lymphocytes (19, 21), it is possible that mTOR-driven proliferation of cells in the TLT may not be a disease-specific process but instead a common pathological process in states of chronic inflammation. The current study opens the door for examining the role of mTOR in TLT formation and growth in other organs and in the cancer microenvironment where TLTs drive disease, predict response to chemotherapy, or complicate outcomes (53). Newer anti-inflammatory therapies may also modulate TLT formation and growth. For example, SGLT2 inhibitors have immunomodulatory and anti-inflammatory effects in the kidney (73). Several studies have shown the effect of SGLT2 inhibitors on T-cell effector function and differentiation via metabolic reprogramming (74). SGLT2 inhibitors can suppress the activation of the NLRP3 inflammasome and inhibit IL-1β and IL-18 (75).

A limitation of TLT studies, in general, is that TLTs cannot be therapeutically targeted without affecting the disease process being studied or systemic immunity. For example, T-cell depletion or dexamethasone treatments that reduce TLTs and improve kidney function (2, 30), or Torin2 as used in the current study, are not specific for TLT depletion. In Pkd1^RC/RC^ mice, the decrease in TLTs with Torin2 was associated with a decrease in cyst growth and an improvement in kidney function (26). However, the effect of Torin2 to decrease cyst growth directly likely also explained the improvement in PKD. Torin2 significantly reduced TLTs in ischemic kidneys but had little effect on tubular injury, possibly because mTORC1 is required for tubular homeostasis and is essential for the tubular response to stress (62).

There are human data that contextualize the findings in the present study. In human transplanted kidneys, VCAM1+ injured proximal tubules surrounded TLTs (16). Age-dependent TLT formation is detectable in the human kidney, and the cellular and molecular components are similar to those in mice, suggesting that age-dependent TLT formation is conserved across species (30). Human TLTs, like mouse TLTs, were composed mainly of T cells, B cells, and follicular dendritic cells that were positive for CXCL13. In human transplanted kidneys, advanced TLTs in protocol biopsies are associated with progressive graft dysfunction (6). Interestingly, pretransplantation rituximab treatment dramatically attenuated the development of stage II TLTs.

In summary, the present study provides novel evidence of intense p-S6 staining in TLTs in four kidney disease models: aged wild-type mice, aged Atg7^−/−^ mice, Pkd1^RC/RC^ mice, and ischemic kidneys. The main evidence supporting a mechanistic role for mTORC1 within TLTs—rather than mTORC1 in the surrounding tubules—in driving TLT formation and growth is the observation that Torin2 significantly reduces cell proliferation, as well as TLT number and size, whereas Raptor knockout in tubules has no significant effect on TLTs.

In conclusion, TLTs play a role in various disease processes across multiple organs, including cancer, infection, autoimmune diseases, transplant rejection, and chronic inflammatory diseases (10). TLTs may be associated with a more favorable course in cancer (53) and infections, but a more severe disease course in autoimmune and chronic inflammatory conditions (10). mTOR inhibitors significantly reduce aging and extend lifespan in mice (76, 77), and are currently used in clinical practice to treat various cancers, transplant rejection, graft restenosis, and tuberous sclerosis complex (78). Newer second-, third-, and fourth-generation mTOR inhibitors are under development (78). The inhibition of TLT development and growth by mTOR inhibitors may have both positive and negative therapeutic implications, which should be carefully considered given their widespread use in medical research and clinical practice.

## Data Availability

The original contributions presented in the study are included in the article/**Supplementary Material**. Further inquiries can be directed to the corresponding author.
