# Supplementary material for: mTOR promotes the formation and growth of tertiary lymphoid tissues in the kidney
Source: Front Immunol. 2025 May 27;16:1527817. doi: 10.3389/fimmu.2025.1527817 (PMC12148856; doi:10.3389/fimmu.2025.1527817)
Supplement: Supplementary file 1 [file DataSheet1.docx]

**Supplement**

**mTOR drives tertiary lymphoid tissue formation and growth in the kidney.**

Daniel J Atwood ^1^, Zhibin He ^1^, Makoto Miyazaki ^2^, Katharina Hopp ^2^, Alkesh Jani^1^, Seth B Furgeson^2^, Sarah Faubel ^2^ and Charles L Edelstein ^1,2^

*1. Rocky Mountain Regional VA Medical Center, 1700 N Wheeling St, Aurora, CO 80045.*

*2. Division of Renal Diseases and Hypertension, University of Colorado Anschutz Medical Campus, 12700 E. 19th Ave, Aurora, CO 80045, United States of America.*

Corresponding Author: Charles L. Edelstein

Correspondence to Charles L. Edelstein, Division of Renal Diseases and Hypertension, Univ. of Colorado at Denver, Box C281, 12700 East, 19th Ave, Aurora, CO 80045, USA.

Phone (303) 724-4810, Fax (303) 724-4868

E-mail: [Charles.edelstein@cuanschutz.edu](mailto:Charles.edelstein@cuanschutz.edu)

Key Words:

Autophagy; mTOR; polycystic kidney; p62.

Supplement Legends


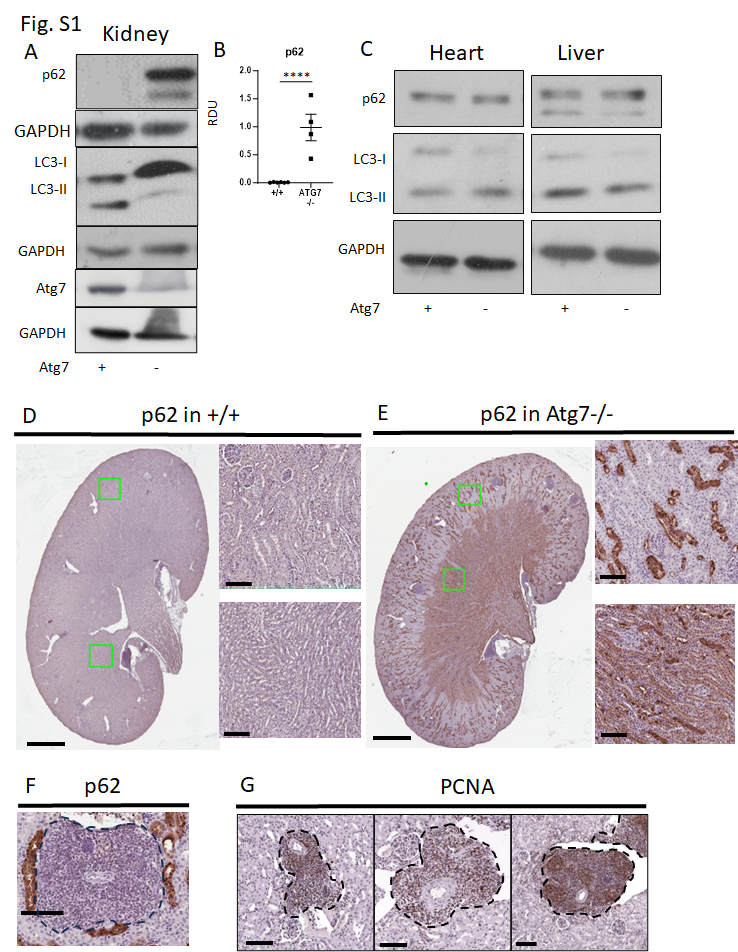


Figure S1: Atg7-/- kidneys have decreased Atg7 and LC3-II and increased p62.

Immunoblot analysis for p62, LC3-I, LC3-II, and Atg7 in wild type and Atg7-/- mice (A). Representative densitometry of immunoblot analysis of p62 in the kidney (B). The increase in p62 was not seen in heart or liver (C). On IHC in +/+ control kidneys there was no staining for p62 (brown) in cortex or medulla. Inserts show high power of staining in cortex and medulla (D). On IHC in Atg7-/- kidneys, there was large staining for p62 (brown) in medulla and less staining in the cortex (E). Inserts show high power of staining in cortex and medulla. Scale bar of inserts = 100µm. There was staining for p62 in the tubules surrounding the TLTs (F). Additional images of PCNA staining (brown) on IHC in TLTs from Atg7-/- kidneys are demonstrated (G). Student’s t-test was used for comparisons between 2 independent groups. A p-value of <0.05 was considered statistically significant. Values are expressed as means ± SEM. ****P<0.0001. Scale bar = 1mm (D,E). Scale bar = 100µm (Inserts, F,G). RDU = relative densitometry units corrected for GAPDH .


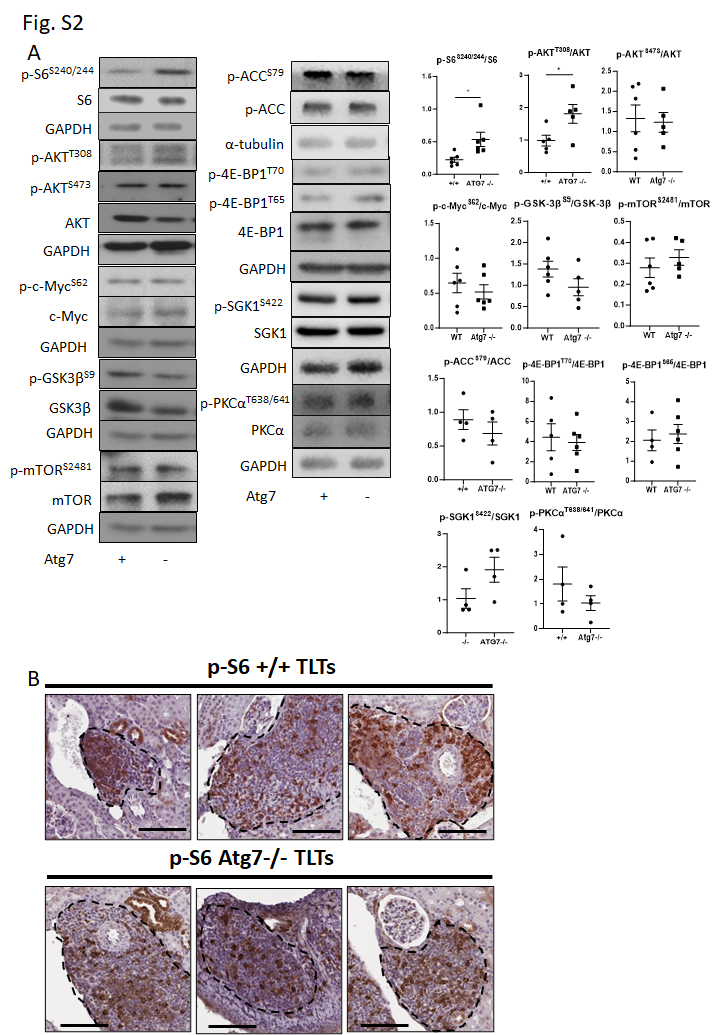


Figure S2: Increased p-S6 and p-Akt^T308^ in Atg7-/- kidneys. No increase in p-ACC ^S79^ , a marker of p-AMPK. No increase in 4E-BP1 isoforms or p-c-myc. No increase in pAkt^S473^, pSGK1^S422^, p-PKCα^T638/641^ p-GSK3β^S9^ or p-mTOR^S2481^, markers of mTORC2. p-S6 staining is present in both +/+ and Atg7-/- kidneys.

Quantitative immunoblot analysis for mTOR proteins in wild type and Atg7-/- kidneys **(A)**. Representative densitometry (relative densitometry units) of immunoblot analysis of proteins in the kidney is demonstrated **(A)**. p-S6 staining (brown) on IHC in TLTs from +/+ and Atg7-/- kidneys is demonstrated **(B)**. Student’s t-test was used for comparisons between 2 independent groups. A p-value of <0.05 was considered statistically significant. Values are expressed as means ± SEM. *P<0.05. Scale bar = 100µm.


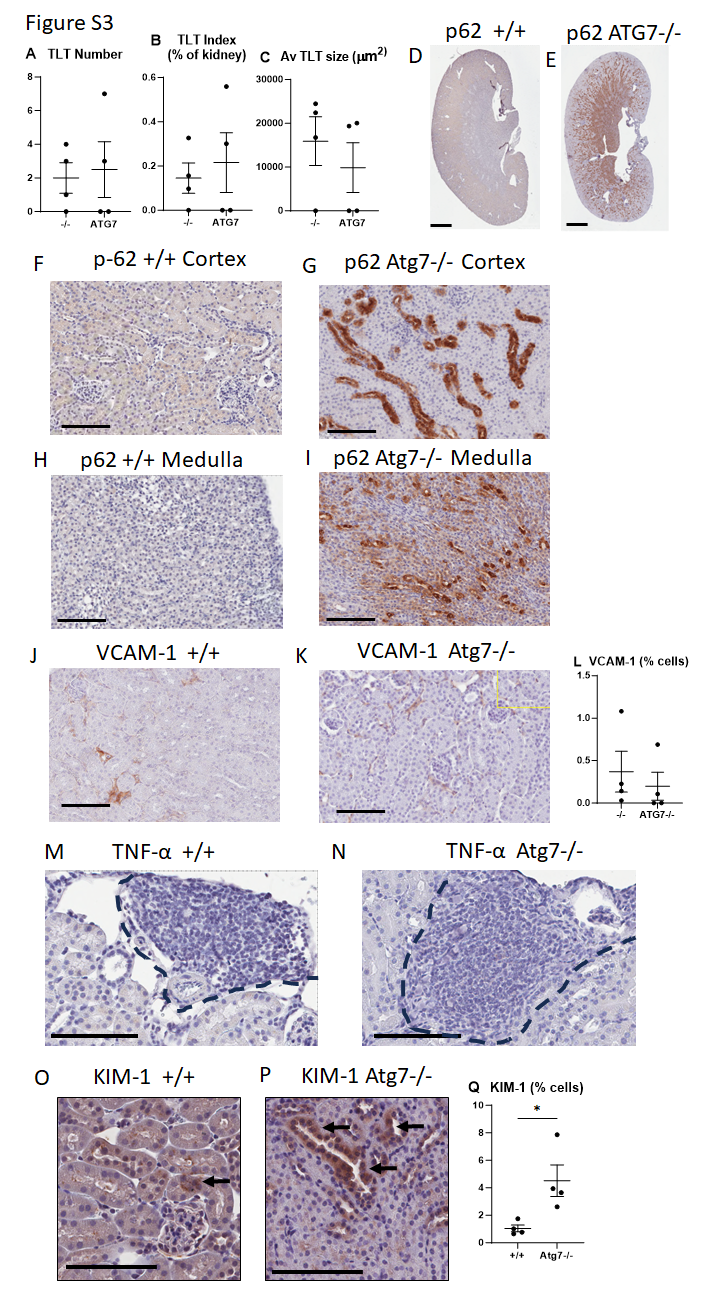


Figure S3. Intense p62 staining in tubules and tubular injury in 180 day old ATG7-/- kidneys. Small TLTs were seen in both wild type and 180 d old ATG7-/- mice **(A, B, C)**. There was intense staining for p62 in tubules in the whole kidney **(D,E)**, cortex **(F,G)** and medulla **(H,I)**. There was little VCAM-1 staining in the ATG7-/- kidneys, that was not localized to tubules surrounding TLTs **(J,K,L)**. There was no staining for TNF-α in the TLTs **(M,N)**. KIM-1 staining (brown, arrows) was increased in ATG7-/- tubules (O,P,Q). Scale bar = 1000 µm (D,E) Scale bar = 100 µm (F-P).


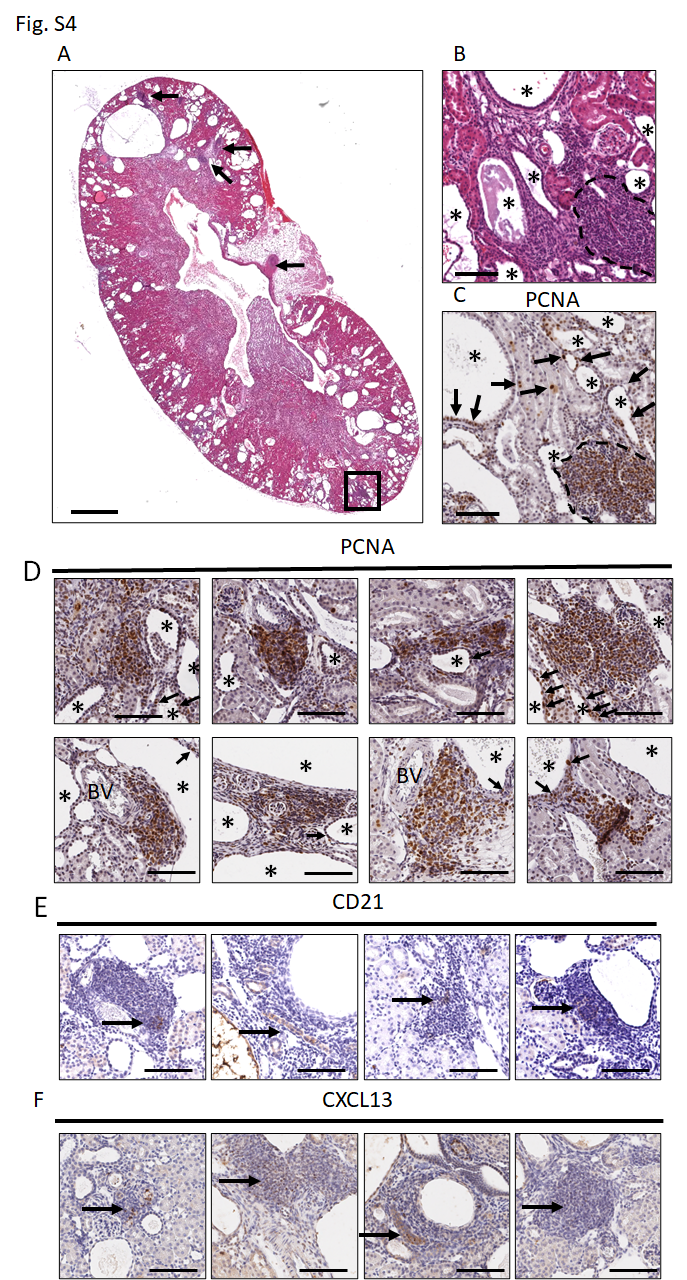


Figure S4: Hematoxylin-eosin, PCNA staining, CD21, and CXCL13 in 120 day old Pkd1^RC/RC^ kidneys

Low power magnification of hematoxylin-eosin staining of TLTs (arrows) in 120 day old Pkd1^RC/RC^ (RC) kidneys **(A)**. Insert showing higher power hematoxylin-eosin staining of TLT (dotted line) in Pkd1^RC/RC^ kidney **(B)**. Insert showing intense PCNA-staining (brown) (dotted line) of TLT and in cells lining cysts (arrows) in Pkd1^RC/RC^ kidney **(C)**. Higher power images of IHC for PCNA (Brown staining) of TLTs in Pkd1^RC/RC^ kidneys **(D)**. There was PCNA staining of cells lining the cysts (arrows). Higher power images of IHC for **(E)** CD21 and **(F)** CXCL13 of TLTs (arrows) in Pkd1^RC/RC^ kidneys show small amounts of staining (Brown staining, arrows). Scale bar = 100 $\mu$m. Scale bar = 1000 µm (A,C). Scale bar = 100 $\mu$m (B,D,E,F,G). Asterisk = cyst. BV = blood vessel.


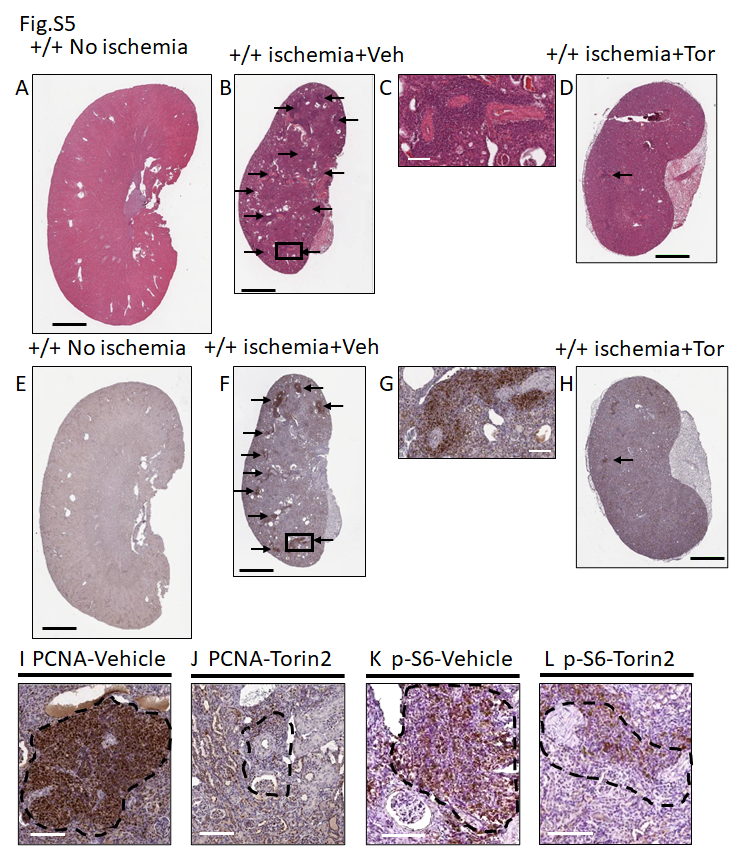


Figure S5: The number and size of TLTs, PCNA and p-S6 staining in TLTs in ischemic kidneys is significantly reduced by the mTOR inhibitor Torin2. Additional images.

TLTs (arrows) were seen on low power and high power magnification of hematoxylin-eosin stained ischemic kidneys compared to no TLTs seen in the contralateral normal kidney **(A, B)**. Ischemic kidneys were smaller than the contralateral control kidney. Insert showing higher power hematoxylin-eosin staining of TLT (square) in ischemic kidney **(C)**. The presence of TLTs (arrows) in ischemic kidneys versus no TLTs in contralateral normal kidneys was confirmed by staining for PCNA **(E, F)**. Insert showing higher power PCNA staining of TLT (square) in ischemic kidney **(G)**. Representative images of hematoxylin-eosin staining and PCNA staining in ischemic kidneys treated with Torin2 showing significantly less TLT number, index and size **(D, H)**. The percentage of cells within the TLTs staining for PCNA was significantly reduced by treatment with Torin2 **(I, J)**. Staining for p-S6 and size of TLTs was significantly reduced by treatment of mice with Torin2 **(K, L)**. Scale bar = 1000 µm (A, B, D, E, F, H). Scale bar = 100µm (C, G, I, J, K, L).


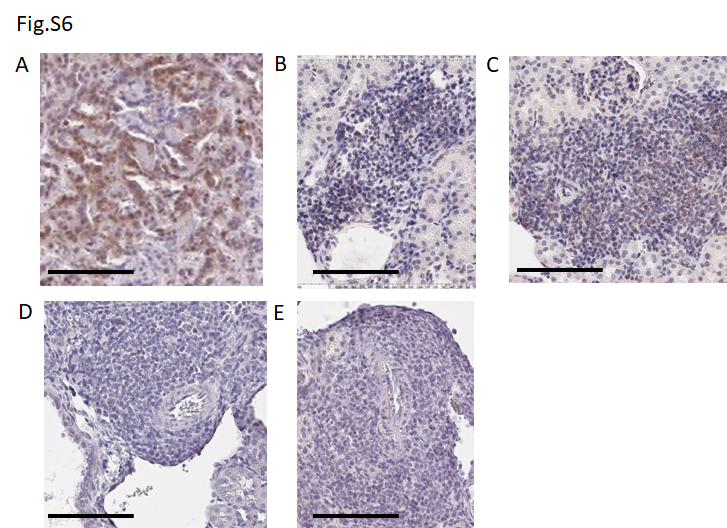


Figure S6: Minimal staining for p-AKT^S473^ (a marker of mTORC2) in TLTs.

TLTs in aged wild type, Atg7-/-, Pkd1^RC/RC^ and ischemia kidneys were stained for p-AKT^S473^. Sections from lung cancer tumors (positive control) showed intense staining (brown) for p-AKT^S473^ **(A)**. There was minimal staining for p-AKT^S473^ in a few of the TLTs in aged wild type **(B)** and aged Atg7-/- **(C)** kidneys. There was no staining for p-AKT^S473^ in any of the TLTs in Pkd1^RC/RC^ **(D)** or ischemia **(E)** kidneys. Scale bar=100µm. N=4 per group. Staining for all 4 groups was performed at the same time in each IHC procedure.


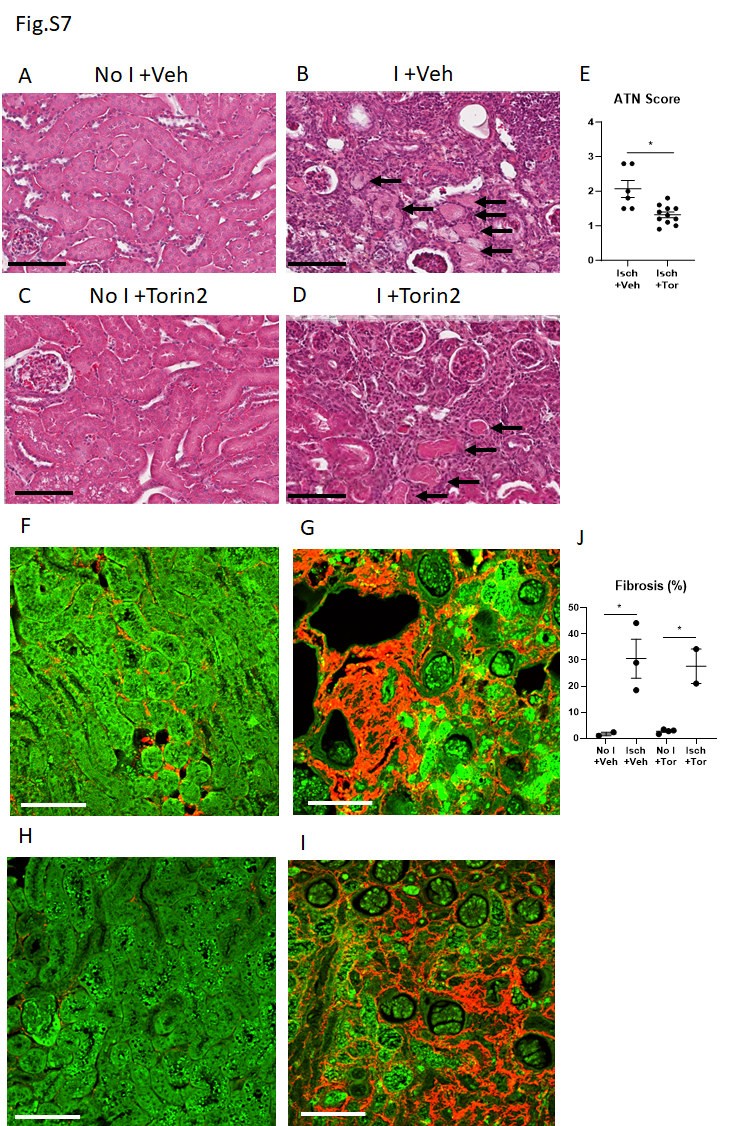


Figure S7: Acute tubular necrosis (ATN) score in ischemia kidneys was reduced by Torin2. Torin2 had no effect on fibrosis in ischemia kidneys.

ATN scores in contralateral non ischemic (No I) and ischemic kidneys (I) treated with vehicle of Torin2 (A-D). Quantitation of ATN scores (E). Fibrosis was determined by second harmonic generation (SHG). Fibrosis (red) in contralateral non ischemic (No I) and ischemic kidneys (I) treated with vehicle or Torin 2 (F-J).

**Supplemental Table 1:** Antibodies used for immunoblot (IB) and immunohistochemistry (IHC) were obtained from Cell Signaling Technology (Danvers, MA), Abcam (Waltham, MA) and Invitrogen (Waltham, MA)

| Antibody | Catalog # | Dilution |
| --- | --- | --- |
| α-tubulin | 2144 | 1:1000 IB |
| AKT | 9272 | 1:1000 IB |
| pAKT^S473^ | 9271 | 1:1000 IB |
| pAKT^S473^ | 4060 | 1:200 IHC |
| pAKT^T308^ | 9275 | 1:1000 IB |
| ACC | 3676 | 1:1000 IB |
| p-ACC^S79^ | 3661 | 1:1000 IB |
| p-cMyc | 13748 | 1:1000 IB |
| cMyc | 5605 | 1:1000 IB |
| CD21 | MA5-32227 | 1:5000 IHC |
| CXCL13 | PA5-86508 | 1:200 IHC |
| GAPDH | 2118 | 1:10,000 IB |
| GSK-3β | 9315 | 1:1000 IB |
| pGSK-3β^S9^ | 9336 | 1:1000 IB |
| LC3-II | 2775 | 1:750 IB |
| mTOR | 2983 | 1:1000 IB |
| pmTOR^S2448^ | 5536 | 1:1000 IB |
| p62 | 5114 | 1:750 IB |
| S6 | 2217 | 1:1000 IB |
| p-S6^S235/236^ | 2211 | 1:1000 IB  1:200 IHC |
| PCNA | 13110 | 1:200 IHC |
| p-SGK1^S422^ | 55281 | 1:1000 IB |
| SGK1 | 59337 | 1:1000 IB |
| VCAM-1 | 32653 | 1:1000 IB  1:200 IHC |
| TNF-α | 3707 | 1:1000 IB  1:200 IHC |
| p-PKCα^T638/641^ | 9375 | 1:1000 IB |
| p-PKCα | 2056 | 1:1000 IB |
| Anti-Rabbit IgG HRP | 7074 | 1:2000 IB |
